# The use of outcome data from quality registries to learn and improve; a Dutch nationwide quantitative analysis in five disease areas

**DOI:** 10.1186/s12913-024-11760-z

**Published:** 2024-10-29

**Authors:** Stacey R. Slingerland, Lise A. M. Moers, Niki M. Medendorp, Paul B. van der Nat, Lineke Derks, Marijke J. C. Timmermans, Nicolette de Keizer, Marc ten Dam, Geke Denissen, Dennis van Veghel

**Affiliations:** 1 Netherlands Heart Registration, Utrecht, the Netherlands; 2 Catharina Heart Centre, Catharina hospital Eindhoven, Eindhoven, the Netherlands; 3 Department of Value Improvement, St. Antonius Hospital, Nieuwegein, the Netherlands; 4 Scientific Centre for Quality of Healthcare, Radboud University Medical Centre, Nijmegen, the Netherlands; 5 National Intensive Care Evaluation, Amsterdam, the Netherlands; 6 Department of Medical Informatics, Amsterdam University Medical Center (location University of Amsterdam), Amsterdam, the Netherlands; 7 Nefrovision, Utrecht, the Netherlands; 8 Cansius-Wilhelmina Hospital, Nijmegen, the Netherlands; 9 Dutch Arthroplasty Register, ‘s-Hertogenbosch, the Netherlands

**Keywords:** Quality improvement, Clinical quality registries, Health outcomes, Outcome monitoring, Healthcare data utilization, Value-based healthcare

## Abstract

**Background:**

Clinical quality registries (CQR) aid in measuring, collecting and monitoring outcome data but it is still unknown how these data are used by hospitals to improve the quality of care. This study assessed the current state of outcome-based quality improvement in the Netherlands in 2022 based on data from multiple disease areas and CQRs; cardiothoracic surgery (Netherlands Heart Registration [NHR]), cardiology (NHR), nephrology, (Nefrovision), intensive care (National Intensive Care Evaluation [NICE]), and orthopaedic surgery (Dutch Arthroplasty Register [LROI]).

**Methods & results:**

The Health Outcomes Management Evaluation (HOME) model was used to assess the current state of outcome-based quality improvement. A questionnaire with 36 questions was sent to healthcare departments of the six disease areas in participating hospitals within five quality registrations in the Netherlands. In total, 124 responses were received; 20 within cardiology, 12 within cardiothoracic surgery, 30 within nephrology, 35 within intensive care and 27 within orthopaedic surgery. Results showed outcome measures were actively used to improve the quality of care, several improvement initiatives were implemented, but outcomes were not always monitored regularly. Results differed between hospitals, but differences were limited between disease areas.

**Conclusion:**

The current state of outcome-based quality improvement in all five disease areas is that outcome measures were consequently employed and used on a frequent basis aiming to achieve quality improvement in healthcare. Results can be improved by structurally embedding the entire improvement cycle into the organisation.

**Supplementary Information:**

The online version contains supplementary material available at 10.1186/s12913-024-11760-z.

## Introduction

Systematically measuring clinical outcomes is essential for improving the quality of care [1]. However, merely measuring outcomes does not guarantee an improvement in care quality; while poor quality leads to poor outcomes, improving quality does not always result in better outcomes. Quality improvement requires the development of a structured approach wherein data can be systematically registered, analysed, reported and integrated [2]. Several models have been developed to aid quality improvement in organisations, such as the plan-do-check-act (PDCA) cycle, which provides a framework for achieving continuous quality improvement [3]. Nonetheless, it remains a challenge to determine how outcome data can be effectively utilized to enhance healthcare practices, and what organizational structures hospitals need to implement these changes.

Clinical quality registries (CQR) serve as valuable tools to collect outcome data, monitor and benchmark process and outcome measures, provide information on efficacy, reduce variation in care, and launch quality improvement initiatives [4, 5]. Worldwide, CQRs such as the SWEDEHEART-clinical registry and the Society of Thoracic Surgeons (STS) National Database, have been demonstrated to contribute to improving the overall quality of care [2, 6–9]. In the Netherlands, several CQRs, such as the Netherlands Heart Registration (NHR), the National Intensive Care Evaluation (NICE), the Dutch Arthroplasty Register (LROI) and Renine registry (Nefrovision), have been established to enhance the transparency and quality of care [10–14].

In 2016, the Health Outcomes Management Evaluation (HOME) model was developed to assess the state of outcome-based quality improvements [15, 16]. The model aimed to gain insight into how data on health outcomes are utilised to improve the quality of care within heart centres of the NHR [5, 15]. The results showed that outcome measures had already been embedded and used within the organisation of healthcare in heart centres for several years, yet significant differences in outcome-based quality improvements existed among these centres, indicating room for improvement. Since the HOME model was previously applied exclusively within the field of cardiology and cardiothoracic surgery, it remains unclear to what extent these earlier findings also extend to other disease areas and CQRs, and whether the results vary between disease areas. Simultaneously, the importance of measuring outcomes has amplified as the Dutch government stimulates the implementation of value-based healthcare [17]. Therefore, it is imperative and currently relevant to evaluate the state of outcome-based quality improvements in Dutch healthcare comprehensively. Hence, the aim of this study is to assess the current state of outcome-based quality improvement using data from CQRs within multiple disease areas and specialisms in the Netherlands in 2022, employing the previously developed HOME model.

## Methods

### Questionnaire

The methodology and questionnaire of the HOME model, as described in Van Veghel et al. (2020) and Van der Nat et al. (2022), were employed in this study. The HOME model consists of a total 11 elements divided into two parts. The first part of the HOME model, known as the outcome-based improvement cycle, exists of four elements and is grounded in Deming’s theory, specifically the PDCA cycle [18]. These elements include: (1) monitoring of outcomes, (2) identification of improvement potential, (3) selection of improvement initiatives and (4) implementation of improvement initiatives. The second part of the HOME model, known as the organizational context, encompasses seven elements, and is based on McKinsey’s 7 S model [19]. These elements include: (1) strategy, (2) governance, (3) culture, (4) leadership, (5) infrastructure, (6) staff and (7) skills. In the HOME model, outcome measures are defined as the indicators that assess the results of a medical treatment, such as mortality, complications, and survival. The questionnaire, totalling 36 questions, was updated and customized compared to the original model as published by Van der Nat et al. (2022). Textual modifications were made, and some answer categories were adjusted to ensure applicability across various disease areas and specialisms. However, no substantial amendments were made. The questionnaire is available in Supplementary Materials 1.

### Study sample

This study included the following specialisms, disease areas and/or interventions with accompanying CQRs:

1. Cardiology: patients undergoing percutaneous coronary intervention (PCI), part of the NHR (from here on mentioned as ‘*cardiology*’);
2. Cardiothoracic surgery: patients undergoing cardiothoracic surgery procedures, part of the NHR (*cardiothoracic surgery*);
3. Intensive care: patients admitted to the intensive care, part of the National Intensive Care Evaluation (NICE) (*intensive care*);
4. Nephrology: patients undergoing dialysis, part of the Renine registry (*nephrology*);
5. Orthopaedic surgery: patients undergoing knee or hip replacement, part of the Dutch Arthroplasty Register (LROI) (*orthopaedic surgery*).

These disease areas and specialisms all collaborate within an umbrella organization called the Samenwerkende Kwaliteitsregistraties (SKR). Between April and June 2022, 261 invitations were emailed to departments in centres participating in the four CQRs. The questionnaires could be completed online. For each disease area, departments were requested to designate at least one physician to complete the survey. Respondents were informed via the invitation that the survey results would be published. Descriptive analyses were conducted using IBM SPSS version 26 and Microsoft Excel.

## Results

A total of 124 out of 261 (47.5%) questionnaires were completed: 20 out of 29 (69.0%) within cardiology, 12 out of 15 (80.0%) within cardiothoracic surgery, 35 out of 72 (48.6%) within intensive care, 30 out of 59 (50.8%) within nephrology and 27 out of 86 (31.4%) within orthopaedic surgery. Results for each element of the HOME model, both for the outcome-based improvement cycle and organizational context, are summarized.

### Outcome-based improvement cycle

Table 1 presents a summary of the results of the outcome-based improvement cycle for each disease area and specialism. For all detailed results, please refer to Supplementary Table 1 in the supplementary materials.

1. **Monitoring of outcomes**: Health outcomes were measured and discussed once a year to quarterly in 80.6% within the department and 62.1% in a multidisciplinary setting, 19.4% and 11.3% respectively, reporting discussion more frequent than quarterly. This varied across disease areas and registrations. For example, intensive care and cardiology discussed outcomes in a multidisciplinary setting less frequently (‘more often than quarterly’ reported by 0.0% and 5.0%, respectively) compared to cardiothoracic surgery (33.3%). In addition, across all registries, department management (47.3%) and the board of directors (22.6%) were involved in the measurement and discussion of outcome measures, while patients (5.9%) were rarely involved. The majority of physicians (median 80.0%) were actively engaged with health outcomes, and 76.6% of the departments utilized additional health outcome data besides information from the CQR. Locally self-developed dashboards and CQR dashboards were often or very often used to discuss health outcomes by 29.8% and 32.3% of respondents, respectively. This usage varied across disease areas and specialisms; for example, locally self-developed dashboards in addition to CQR dashboards were more frequently used in cardiothoracic surgery (50.0%) than in intensive care (8.6%).
2. **Identification of improvement potential**: Analyses to identify improvement potential within all disease areas and specialisms were often or very often performed at hospital level, both risk-unadjusted (54.0%) and risk-adjusted (45.2%). Analyses on inter-doctor variation in risk-unadjusted (12.1%) and risk-adjusted (8.1%) health outcomes were less common. When comparing disease areas and specialisms, nephrology and cardiothoracic surgery overall utilized these analyses more than intensive care and orthopaedic surgery (the questions regarding inter-doctor variation were not applicable for intensive care). On average, 44.4% of all respondents set targets for one or some health outcomes. In 10.5% of the improvement initiatives, the initiative was started after outcome reports revealed that the hospital was performing significantly worse than the average of other hospitals. In contrast, 69.4% of the initiatives were started after outcome reports led to clinically relevant insights.
3. **Selection of improvement initiatives**. Additional analyses have been conducted on average for two (median 2.0) outcome measures, and two (median 2.0) improvement initiatives per centre have been initiated by monitoring outcomes in the past two years. The learning strategies considered successful in identifying improvement initiatives that were often or very often used included internal peer sharing of knowledge, experience or techniques (62.1%), scientific literature (61.3%) and file review (56.5%), while consultation of external experts and structural learning environment with other hospitals were utilised less frequently (8.9% and 21.8%, respectively). Overall, nephrology and cardiothoracic surgery employed learning strategies most often, while cardiology used them the least frequently compared to the other disease areas and specialisms.
4. **Implementation of improvement initiatives**. On average, both the implementation and effects of improvement initiatives were monitored to some extent in all disease areas and specialisms (medians 7.0 on a scale of 0 to 10). Orthopaedic surgery achieved the highest scores (medians 7.0 and 8.0), while cardiology had the lowest (median 5.5 and 6.0).

**Table 1:** Results per quality registration for each element of the outcome-based improvement cycle

Question	Answer options	Cardiology	Cardiothoracic surgery	Intensive care	Nephrology	Orthopeadic surgery	Total
Outcome-based improvement cycle		*N* = 20	*N* = 12	*N* = 35	*N* = 30	*N* = 27	*N* = 124
**1. Monitoring of outcomes**							
How frequent are health outcomes measured and discussed within the department?	Less than once a year to quarterly	16 (80.0%)	8 (66.7%)	30 (85.7%)	28 (93.3%)	18 (66.7%)	100 (80.6%)
	More often than quarterly	4 (20.0%)	4 (33.3%)	5 (14.3%)	2 (6.7%)	9 (33.3%)	24 (19.4%)
How frequent are health outcomes measured and discussed in a multidisciplinary setting?	Less than once a year to quarterly	10 (50.0%)	6 (50.0%)	20 (57.1%)	25 (83.3%)	16 (59.3%)	77 (62.1%)
	More often than quarterly	1 (5.0%)	4 (33.3%)	0 (0.0%)	3 (10.0%)	6 (22.2%)	14 (11.3%)
Which other parties within your hospital, apart from the multidisciplinary meetings or department meetings, are involved in the measurement and discussion of outcome measures? (m*ultiple answers possible)*	Patients	0 (0.0%)	0 (0.0%)	0 (0.0%)	9 (10.7%)	5 (8.5%)	14 (5.9%)
	Department management	14 (56.0%)	7 (38.9%)	26 (49.1%)	32 (38.1%)	34 (57.6%)	113 (47.3%)
	Board of directors	3 (12.0%)	6 (33.3%)	12 (22.6%)	19 (22.6%)	14 (23.7%)	54 (22.6%)
How many physicians that are directly involved in the treatment of patients are involved in the discussion of outcome measures? (*Median (IQR))*		10.5 (6.0-20.75)	14.0 (6.5-29.75)	11.0 (6.0–23.0)	7.5 (5.0–15)	6.0 (5.0–9.0)	9.0 (5.0-17.5)
How many physicians take active knowledge of health outcomes of your hospital? (*Median (IQR))*		90.0 (50.0-100)	80.0 (55.0-97.5)	50.0 (11.0-100.0)	77.5 (33.75–100)	100 (8.0-100)	80.0 (20.0-100)
How frequent are (digital) dashboards, in which health outcomes are displayed, used to discuss health outcomes within your speciality? (*results shown for the combined answer options of ‘often’ and ‘very often’*)	Self developed dashboards	5 (25.0%)	6 (50.0%)	3 (8.6%)	12 (40.0%)	11 (40.7%)	37 (29.8%)
	CQR dashboards	4 (20.0%)	4 (33.3%)	11 (31.4%)	12 (40.0%)	9 (33.3%)	40 (32.3%)
Does your department use additional health outcomes besides information from the quality registry?	Yes	12 (60.0%)	10 (83.3%)	21 (60.0%)	27 (90.0%)	25 (92.6%)	95 (76.6%)
**2. Identification of improvement potential**							
To what extent are the following analyses performed to identify improvement potential? (*results shown for the combined answer options of ‘often’ and ‘very often’*)	Unadjusted health outcomes of your hospital	10 (50.0%)	10 (83.3%)	11 (31.4%)	22 (73.3%)	14 (51.8%)	67 (54.0%)
	Risk-adjusted health outcomes of your hospital	9 (45.0%)	6 (50.0%)	11 (31.4%)	18 (60.0%)	12 (44.4%)	56 (45.2%)
	Unadjusted health outcomes compared to other hospitals	5 (25.0%)	4 (33.3%)	7 (20.0%)	9 (30.0%)	5 (18.5%)	30 (24.2%)
	Risk-adjusted health outcomes compared to other hospitals	6 (30.0%)	6 (50.0%)	8 (22.9%)	10 (33.3%)	3 (11.1%)	33 (26.6%)
	Inter-doctor variation in unadjusted health outcomes	2 (10.0%)	3 (25.0%)	n/a	6 (20.0%)	4 (14.8%)	15 (12.1%)
	Inter-doctor variation in risk-adjusted health outcomes	1 (5.0%)	3 (25.0%)	n/a	4 (13.3%)	2 (7.4%)	10 (8.1%)
Have targets been set for outcomes measures in the last two years within your department?	Yes, for one or some health outcomes	10 (50.0%)	4 (33.3%)	17 (48.6%)	12 (40.0%)	12 (44.4%)	55 (44.4%)
	Yes, for one or some health outcomes and for at least one of the medical conditions for which outcomes are available	2 (10.0%)	3 (25.0%)	0 (0.0%)	4 (13.3%)	7 (25.9%)	16 (12.9%)
	Yes, for all health outcomes	0 (0.0%)	0 (0.0%)	3 (8.6%)	6 (20.0%)	3 (11.1%)	12 (9.7%)
When have outcome reports led to improvement initiatives in your hospital in the past 2 years? *(multiple answers possible)*	If the hospital has significant less favourable outcomes than the average.	6 (30.0%)	5 (41.7%)	11 (31.4%)	14 (46.7%)	9 (33.3%)	45 (36.3%)
	If the reporting leads to clinically relevant insights that can be a starting point for improvements	13 (65.0%)	9 (75.0%)	24 (68.6%)	23 (76.7%)	17 (63.0%)	86 (69.4%)
	If other hospitals have more favourable outcomes.	1 (5.0%)	3 (25.0%)	2 (5.7%)	6 (20.0%)	1 (3.7%)	13 (10.5%)
**3. Selection of improvement initiatives**							
For how many outcome indicators have additional analyses been carried out in the past two years? (*Median (IQR))*		2.0 (1.0–3.0)	3.0 (2.0–4.0)	2.0 (0.0–5.0)	3 (2.0–4.0)	3.0 (2.0–4.0)	2.0 (1.0–4.0)
How many improvement initiatives have been initiated by monitoring outcomes of care in the past two years? (*Median (IQR))*		1.5 (1.0-2.75)	2 (1.25–4.75)	2.0 (1.0–3.0)	3.0 (2.0-5.25)	3.0 (2.0–5.0)	2.0 (1.0–4.0)
To what extent are the following learning strategies proved to be successful to create improvement initiatives within the organisation? (*results shown for the combined answer options of ‘often ’and ‘very often’*)	Best practice	2 (10.0%)	3 (25.0%)	7 (20.0%)	9 (30.0%)	10 (37.0%)	31 (25.0%)
	Process analysis	3 (15.0%)	2 (16.7%)	7 (20.0%)	12 (40.0%)	10 (37.0%)	34 (27.4%)
	File analysis of patients	10 (50.0%)	8 (66.7%)	21 (60.0%)	15 (50.0%)	16 (59.3%)	70 (56.5%)
	Scientific literature	11 (55.0%)	8 (66.7%)	23 (65.7%)	17 (56.7%)	17 (63.0%)	76 (61.3%)
	Guidelines studied and implemented more rigorously	8 (40.0%)	6 (50.0%)	19 (54.3%)	19 (63.3%)	15 (55.6%)	67 (54.0%)
	Initiatives based on clinical experience	9 (45.0%)	7 (58.3%)	11 (31.4%)	18 (60.0%)	13 (48.1%)	58 (46.8%)
	Structural learning environment with other hospitals	5 (25.0%)	2 (16.7%)	5 (14.3%)	7 (23.3%)	8 (29.6%)	27 (21.8%)
	Consultation of external experts	1 (5.0%)	1 (8.3%)	2 (5.7%)	7 (23.3%)	0 (0.0%)	11 (8.9%)
	Internal peer sharing of knowledge, experience or techniques	10 (50.0%)	5 (41.7%)	18 (51.4%)	25 (83.3%)	19 (70.4%)	77 (62.1%)
**4. Implementation of improvement initiatives**							
To what extent is the implementation of improvement initiatives monitored? (*Median (IQR); scale of 0 to 10*)		5.5 (3.5-7.0)	6.5 (3.5-8.0)	6.0 (5.0–8.0)	7.0 (6.0–8.0)	7.0 (6.0–8.0)	7.0 (5.0–8.0)
To what extent is the effect of improvement initiatives monitored? (*Median (IQR); scale of 0 to 10*)		6.0 (3.0–7.0)	7.5 (6.25–8.75)	6.0 (5.0–8.0)	7.0 (6.0–8.0)	8.0 (6.0–8.0)	7.0 (6.0–8.0)

This table shows the summary of the results in which some answers are merged. All results and all answer categories are displayed in Supplementary Table 1 in the Supplementary materials

*N/a *not applicable, *CQR *clinical quality registration

### Organizational context

Table 2 provides a summary of the results of the seven elements regarding the organizational context, stratified for each disease area and specialism. For all detailed results, please refer to Supplementary Table 1 in the supplementary materials.

1. **Strategy.** The measurement and improvement of outcomes were explicitly included in the (multi)annual plan of the specialism (40.9%), department (28.9%) and hospital (23.5%). Results for the hospital’s (multi)annual plan were quite similar across disease areas and specialisms (ranging from 18.2 to 28.2%), while results differed regarding the (multi)annual plan of the specialism (ranging from 17.5% for nephrology to 65.0% for intensive care) and department (ranging from 5.0 to 55.6%).
2. **Governance.** In general, 35.0% of respondents stated that no external parties were involved in the measurement and discussion of health outcomes. The most commonly involved external parties were networks with other hospitals (40.1%), referring hospitals (10.9%), and general practitioners (5.1%). Cardiothoracic surgery and cardiology showed the highest percentages regarding involving referring hospitals in the measurement and discussion of health outcomes (53.8% and 22.7% respectively) compared to a range of 2.8–3.0% for other disease areas and specialisms.
3. **Culture**. Within their specialism, respondents experienced trust in openly discussing aggregated outcomes (median 8.0 on a scale of 0 to 10), outcomes by operator (8.0), and aggregated outcomes between specialisms (8.0). However, respondents generally deemed it important to improve the quality of care based on outcome indicators (8.0 on a scale of 0 to 10). Overall, nephrology and orthopaedic surgery reported the highest trust (8.0 to 9.0) while cardiology experienced the lowest (6.5 to 8.0).
4. **Leadership.** The job positions that most often had a leadership role in achieving an improvement cycle based on outcome indicators were physicians (65.3%), followed by management (21.0%). Cardiology and cardiothoracic surgery were the only disease areas and specialisms that stated that nurses and management were not involved (0.0%). Additionally, 81.5% of all respondents stated that no other level within the organization took a leading role in achieving an improvement cycle.
5. **Infrastructure.** On average, 80.0% of data from quality registration was recorded during regular care in the electronic health record (EHR), and 76.6% of data was submitted partly or entirely to the quality registry via the EHR. Data quality was most often checked by a data manager (48.4%) or physician (33.1%), and majority of respondents (54.0%) reported uploading data to the registry less than quarterly. On average, respondents rated their trust in the data quality at 8.0 (median, on a scale of 0 to 10), while data from other hospitals received a lower rating (median 7.0). Cardiothoracic surgery reported the highest trust compared to other disease areas and specialisms (median 8.5 versus 8.0anging from 7.0 to 8.3).
6. **Staff.** Medical specialists (35.5%) and quality managers (27.0%) were most often provided with time to implement improvement cycles. On average, 4.0 h per week were made available for this particular task, with intensive care and cardiology reporting the lowest number of hours per week (median 2.0), while nephrology had the highest (median 6.0).
7. **Skills**. On average, 2.0 (median) physicians within the department had clear expertise with and affinity for data management and data analytics. Cardiothoracic surgery had the highest number (median 2.5) compared to the other disease areas (median 2.0).

**Table 2:** Results per quality registration for each element of the organizational context

Organizational context	Answer options	Cardiology	Cardiothoracic surgery	Intensive care	Nephrology	Orthopaedic surgery	Total
		*N* = 20	*N* = 12	*N* = 35	*N* = 30	*N* = 27	*N* = 124
**1. Strategy **							
Is the measuring and improving of outcomes of the delivered care, using outcome indicators explicitly, part of the (multi)annual plan of one of the following internal policies? *(multiple answers possible)*	Specialism’s	15 (38.5%)	5 (22.7%)	39 (65.0%)	11 (17.5%)	29 (50.0%)	99 (40.9%)
	Departments	8 (20.5%)	12 (54.5%)	3 (5.0%)	35 (55.6%)	12 (20.7%)	70 (28.9%)
	Hospital-wide	11 (28.2%)	4 (18.2%)	14 (23.3%)	14 (22.2%)	14 (24.1%)	57 (23.5%)
**2. Governance**							
Which parties within the entire chain of care delivery, besides the parties involved in your own hospital, are involved in the measurement and discussion of health outcomes? (m*ultiple answers possible)*	None	8 (36.4%)	5 (38.5%)	10 (27.8%)	14 (42.4%)	11 (33.3%)	48 (35.0%)
	Referring hospitals	5 (22.7%)	7 (53.8%)	1 (2.8%)	1 (3.0%)	1 (3.0%)	15 (10.9%)
	Network or collaboration with other hospitals	8 (36.4%)	1 (7.7%)	23 (63.9%)	13 (39.4%)	10 (30.3%)	55 (40.1%)
	General practitioners	1 (4.5%)	0 (0.0%)	0 (0.0%)	1 (3.0%)	5 (15.2%)	7 (5.1%)
**3. Culture**							
In your opinion, how important is improving quality of care based on outcome indicators considered within your specialism? (*Median (IQR); scale of 0 to 10*)		7.0 (6.0–8.0)	8.5 (7.25–9.75)	7.0 (6.0–8.0)	8.0 (7.0–8.0)	8.0 (7.0–8.0)	8.0 (7.0–8.0)
In your opinion, how high is the trust to openly talk about… (*Median (IQR); scale of 0 to10*)	Aggregated outcomes within your specialism?	8.0 (8.0–9.0)	8.0 (8.0–9.0)	9.0 (8.0–10)	9.0 (8.0–9.0)	9.0 (7.0–9.0)	8.0 (8.0–9.0)
	Outcomes by operator within your specialism?	7.0 (6.0–8.0)	8.0 (7.0–9.0)	n/a	8.0 (7.0–9.0)	9.0 (7.0–9.0)	8.0 (7.0–9.0)
	Aggregated outcomes between specialisms?	6.5 (5.0–8.0)	8.0 (7.0–9.0)	8.0 (7.0–9.0)	8.0 (7.0–9.0)	7.0 (6.0–9.0)	8.0 (6.0–8.0)
** 4. Leadership**							
Different levels within an organisation are shown below. Please rank the levels in order of the extent to which a leadership role is taken to achieve an improvement cycle based on outcome indicators? (*highest priority*)	Physicians	16 (80.0%)	12 (100.0%)	17 (48.6%)	16 (53.3%)	20 (74.1%)	81 (65.3%)
	Nurses	0 (0.0%)	0 (0.0%)	0 (0.0%)	2 (6.7%)	1 (3.7%)	3 (2.4%)
	Management	0 (0.0%)	0 (0.0%)	15 (42.9%)	8 (26.7%)	3 (11.1%)	26 (21.0%)
Is there another level within the organisation which takes a leading role to realise an improvement cycle based on outcome indicators?	Yes	4 (20.0%)	4 (33.3%)	3 (8.6%)	4 (13.3%)	8 (29.6%)	23 (18.5%)
	No	16 (80.0%)	8 (66.7%)	32 (91.4%)	26 (86.7%)	19 (70.4%)	101 (81.5%)
If yes, which level?	Quality department	4 (100.0%)	2 (50.0%)	1 (33.3%)	4 (100%)	0 (0.0%)	11 (47.8%)
	Research & Development	0 (0.0%)	1 (25.0%)	0 (0.0%)	0 (0.0%)	2 (25.0%)	3 (13.0%)
**5. Infrastructure**							
What percentage of data from the quality registration is recorded during regular care in the EHR? (*Median (IQR)*)		90.0 (76.3–95.0)	82.3 (75.0-92.5)	80.0 (50.0–90.0)	75.0 (20.0–90.0)	80.0 (30.0–99.0)	80.0 (50.0–95.0)
How does the data get to the quality registry?	Manual input into quality registration application (retyping)	1 (5.0%)	0 (0.0%)	3 (8.6%)	7 (23.3%)	7 (25.9%)	18 (14.5%)
	Upload of template filled partly or entirely by extraction from EHR	17 (85.0%)	12 (100%)	31 (88.6%)	19 (63.3%)	16 (59.2%)	95 (76.6%)
How is the data checked for quality before delivery?	By a data manager	16 (80.0%)	9 (75.0%)	15 (42.9%)	9 (30.0%)	11 (40.7%)	60 (48.4%)
	By a physician	3 (15.0%)	3 (25.0%)	15 (42.9%)	11 (36.7%)	9 (33.3%)	41 (33.1%)
How often are data uploaded to the quality registry?	Less than once a year to quarterly	16 (80.0%)	10 (83.3%)	9 (25.7%)	21 (70.0%)	11 (40.7%)	67 (54.0%)
	More often than quarterly	2 (10.0%)	2 (16.7%)	26 (74.3%)	3 (10.0%)	11 (40.7%)	44 (35.5%)
How high is your trust in the quality of data from your own hospital from the quality registration? *(Median (IQR); scale of 0 to 10)*		8.0 (8.0–9.0)	8.5 (8.0–9.0)	8.0 (7.0–9.0)	8.0 (8.0–9.0)	8.0 (7.0–9.0)	8.0 (7.0–9.0)
How high is your trust in the quality of data from other hospitals from quality registration? *(Median (IQR); scale of 0 to 10)*		7.0 (7.0–8.0)	7.0 (6.0–8.0)	7.0 (6.0–8.0)	7.0 (7.0–8.0)	7.0 (5.0–8.0)	7.0 (6.0–8.0)
**6. Staff**							
Which of the following officer(s) are provided with time to implement an improvement cycle aimed at improving outcomes of care?	Medical specialists	10 (43.5%)	8 (57.1%)	12 (30.0%)	12 (27.9%)	12 (37.5%)	54 (35.5%)
	Quality managers	7 (30.4%)	3 (21.4%)	7 (17.5%)	16 (37.2%)	8 (25.0%)	41 (27.0%)
In total, how many hours per week do these individuals have available of this? (*Median (IQR)*)		2.0 (1.0-4.75)	3.0 (1.25–4.75)	2.0 (1.0–6.0)	6.0 (4.0–16.0)	4.0 (2.0–10.0)	4.0 (2.0–8.0)
**7. Skills**							
How many doctors are there in the hospital with clear expertise and affinity for data management and data analytics? (*Median (IQR)*)		2.0 (2.0–3.0)	2.5 (2.0–3,75)	2.0 (1.0-2.5)	2.0 (1.0–2.0)	2.0 (1.0–3.0)	2.0 (1.0–3.0)

This table shows the summary of the results in which some answers are merged. All results and all answer categories are displayed in Supplementary Table 1 in the Supplementary materials

*N/a* not applicable, *EHR *electronic health record

## Discussion

In this study, the Health Outcome Management Evaluation (HOME) model was used to assess the current state of outcome-based quality improvement using data from CQRs within multiple disease areas and specialisms in the Netherlands. In 2022 results were regularly monitored, improvement potential was identified using different types of analyses, and almost half of the respondents stated at least one or some health outcomes targets had been set in the past two years. All respondents agreed that improving the quality of care is important and frequently used and discussed outcome information within their departments. Measuring and improving outcomes were commonly part of the specialism’s (multi)annual plan. In addition, there was moderate trust in discussing aggregated outcomes within and between specialisms, although varying by disease area and specialism. Still, results showed there is room for improvement. The implementation of improvement initiatives and the effect of the implementation were not always monitored well and the involvement of stakeholders, such as patients and management was limited. In addition, the number of improvement initiatives, on average two projects in two years, might have been higher if the COVID-19 pandemic did not severely impact the day-to-day operations [20].

The majority of the elements in the model showed similar scores across the different disease areas and specialisms, although there were some differences due to the nature of the disease area and specialism. For example, cardiology had fewer multidisciplinary discussions on health outcomes compared to other disease areas and specialisms due to its more monodisciplinary nature. Cardiothoracic surgery had the highest percentage of outcome discussions with referring hospitals, explained by the Dutch organisation of cardiac surgery by a hub-spoke model [21–23]. Furthermore, all disease areas and specialisms, except intensive care, primarily used self-developed dashboards. The reasons for underutilization of CQR dashboards in hospitals remain unclear. Possible explanations include a lack of expertise in their utilization and the limited applicability of national CQRs to local contexts. Another explanation found in literature is that CQRs provide consensus-based health outcome indicators, but hospitals may also track additional indicators not covered by CQRs due to variances in care pathways [5]. Future research should explore the alignment and differences in indicators and health outcomes between self-developed and CQR dashboards and how CQRs can adjust their dashboards to overcome these differences.

The elements of the outcome-based improvement cycle and organisational context were not uniformly implemented. The monitoring of health outcomes and identification of improvement potential (elements 1 and 2) were predominant while the selection and implementation of improvement initiatives (3 and 4) were less present. This aligns with literature suggesting PDCA cycles are often aborted prematurely when the process is not clear or it is found to be ineffective [3]. One possible solution to address this challenge is the integration of other methodologies like Lean or Six Sigma within the PDCA cycle, which can provide additional tools and frameworks for utilizing data effectively to drive further improvement. Realising quality improvement may entail structurally embedding the entire improvement cycle. A recommendation for future research is to study the reasons behind the varied application of the elements of the HOME model and whether there is a role for CQRs to support hospitals in balancing these elements.

Regarding the organisational context, this study showed that primarily physicians drove the realisation of improvement cycles, often without involvement from management or the hospital board. While measuring and improving outcomes were often part of the specialism’s (multi)annual plan, integration into hospital-wide plans was lacking. Nonetheless, leadership is a crucial factor in achieving good coordination of quality measures and facilitating the implementation of improvement initiatives [24, 25]. Although physician leadership and physician-driven initiatives have been emphasized previously, value-based healthcare (VBHC) highlights that all stakeholders need to prioritise improvement in quality, or patient value, as an overarching goal [16, 26]. Further progress in implementing outcome measures in the organisation could be achieved through advancing Integrated Practice Units (IPUs), i.e. organising of care for a certain patient group, and prioritizing health outcome improvement at all levels of management within healthcare [26].

The survey revealed variations in the level of confidence in data quality, data upload to the CQR and registration burden. Respondents generally trusted the quality of data originating from their hospital more than data from other hospitals. Disease areas and specialisms like cardiothoracic surgery and cardiology, where data managers checked the quality of the data and the majority of data was uploaded from the EHR to the CQR, experienced high trust in the quality of the data across hospitals. To the best of our knowledge, there are no other studies on physician’s trust in data in CQRs. Additionally, nearly 70% of quality registration data was recorded during regular care activities, indicating a significant portion of healthcare professionals’ time spent on registering quality indicators. This aligns with two recent studies that pointed out that on average, a healthcare professional in the Netherlands spends almost an hour per working day registering quality indicators, leading to registration burden and reducing intrinsic motivation and time spent on patient care [27, 28]. Decreasing registration burden requires improving operational efficiency in data collection, such as better administrative support or integrating care pathways [28]. Direct EHR-to-CQR data upload could reduce the registration burden and free up time for data analysis and implementing quality improvement efforts.

Finally, this study extends a previous study conducted in 2020, which focused on outcome-based quality improvement in cardiothoracic surgery and cardiology departments [15]. Comparing the results of the two studies could offer valuable information into the evolution within these disease areas and specialisms over time. While the current study included a larger number of heart centers (32 compared to 20), the findings regarding outcome-based quality improvement were similar, without a clear trend observed. The reasons for this lack of improvement compared to previous studies are unclear; it could be due to a shift in prioritization during the COVID-19 pandemic. Future research should consider repeating this study across all five disease areas and specialisms to monitor and track development and improvements in outcomes.

### Limitations

This study faces several limitations. First, there is a wide response range across disease areas (30.0-80.0%), potentially limiting the representation of each disease area. Furthermore, it is unknown what the sizes of the departments of the respondents who completed the survey were. Second, results may not fully represent the entire CQR for the NHR and LROI as only one condition from each specialism is included, while the specialisms have multiple conditions and patient groups. Third, the self-reported nature of the questionnaire may introduce socially desirable responses [29]. Fourth, there could be selection bias, with more interested and experienced respondents being more likely to participate, potentially overestimating the current state of outcome-based quality improvement in the Netherlands. Fifth, despite the survey originates from two previously published studies, some questions leave room for interpretation, limiting the analysis of the results. Additionally, while the study focused on the extent and utilization of health outcomes, the effectiveness of their use remains unclear, suggesting a need for future studies to address this aspect. Last, due to the quantitative nature of this study, there are inherent limitations to the depths of analysis and interpretation of the results. Conducting qualitative research could offer a better understanding of the underlying significance of the study results. It could provide deeper insights into the various factors influencing quality improvement initiatives and help explain the differences observed across disease areas.

## Conclusion

This study assessed the current state of outcome-based quality improvement in the Netherlands in 2022 across multiple disease areas and specialisms. Outcome measures within these disease areas are utilised to drive quality improvement efforts, as outcomes are monitored, discussed and used to initiate new improvement initiatives. Realising improvement may entail structurally embedding the entire improvement cycle, increasing the frequent usage of outcome measures in healthcare, and enhancing the involvement of management and leaders.

## Electronic supplementary material

Below is the link to the electronic supplementary material.

Supplementary Material 1.

Supplementary Material 2.

## Data Availability

The data that support the findings of this study are not publicly available but are available on request from the corresponding author [DvV].

## Abbreviations

CQR: Clinical quality registry
DHD: Dutch Hospital Data
EHR: Electronic health record
HOME: Health Outcomes Management Evaluation
LROI: Dutch Arthroplasty Registry
NHR: Netherlands Heart Registration
NICE: National Intensive Care Evaluation
PCI: Percutaneous coronary intervention
PDCA: Plan-do-check-act
SKR: Samenwerkende Kwaliteitsregistraties
STS: Society of Thoracic Surgeons
