# Supplementary material for: The use of outcome data from quality registries to learn and improve; a Dutch nationwide quantitative analysis in five disease areas
Source: BMC Health Serv Res. 2024 Oct 29;24:1296. doi: 10.1186/s12913-024-11760-z (PMC11520663; doi:10.1186/s12913-024-11760-z)
Supplement: Supplementary file 1 — Supplementary Material 1. [file 12913_2024_11760_MOESM1_ESM.docx]

**Health Outcome Management Evaluation (HOME) model – QUESTIONAIRE**

**1.On behalf of which centre is this survey being completed?**

[open: text field]

**2. Please not the positions of the people who helped complete this survey.**

[open: text field]

**3. How frequent are health outcomes measured and discussed within the department?**

Not

Less than once a year

Once a year

Between once a year and quarterly

Quarterly

Between quarterly and monthly

Monthly

More often than monthly

Unknown

**4. How frequent are health outcomes measured and discussed in a multidisciplinary setting?**

Not

Less than once a year

Once a year

Between once a year and quarterly

Quarterly

Between quarterly and monthly

Monthly

More often than monthly

Unknown

**5. Which other parties within your hospital, apart from the multidisciplinary meetings or department meetings, are involved in the measurement and discussion of outcome measures? *Multiple answers possible***

None

Patients

Hospital management

Staff board

Board of directors

Other

**6. Which parties within the entire chain of care delivery, besides the parties involved in your own hospital, are involved in the measurement and discussion of health outcomes? *(Multiple answers possible)***

None

Referring hospitals

Network or collaboration with other hospitals

General practitioners

Nursery homes

Revalidation

Domestic care

Mental health services

Health insurer

Other

**7. How many physicians that are directly involved in the treatment of patients are involved in the discussion of outcome measures?**

[open: numeric field]

**8. How many physicians take active knowledge of health outcomes of your hospital?**

[open: 0%-100%]

**9. How frequent are (digital) dashboards, in which health outcomes are displayed, used to discuss health outcomes within your speciality?**

**Self-developed dashboards**

Never

Rarely

Sometimes

Often

Very often

**CQR dashboards**

Never

Rarely

Sometimes

Often

Very often

**10. Does your apartment use additional health outcomes besides information from the quality registry?**

Yes

No

Don't know/other

**11. How frequent are the following types of analyses performed to identify improvement potential?**

**Uncorrected health outcomes of your hospital**

Never

Rarely

Sometimes

Often

Very often

**Risk-corrected health outcomes of your hospital**

Never

Rarely

Sometimes

Often

Very often

**Uncorrected health outcomes compared to other hospitals**

Never

Rarely

Sometimes

Often

Very often

**Risk-corrected health outcomes compared to other hospitals**

Never

Rarely

Sometimes

Often

Very often

**Inter-doctor variation in uncorrected health outcomes**

Never

Rarely

Sometimes

Often

Very often

**Inter-doctor variation in risk-corrected health outcomes**

Never

Rarely

Sometimes

Often

Very often

**12 For how many outcome indicators have additional analyses been carried out in the past two years at the initiative or request of your specialism with the aim of better interpreting results and possible arriving at improvement initiatives?**

[open: numeric field]

**13. Have targets been set for outcomes measures in the last two years within your department?**

No

Yes, for one or some health outcomes

Yes, for one or some health outcomes and for at least one of the medical conditions for which outcomes are available

Yes, for all health outcomes

Other

**14. Is the measuring and improving of outcomes of the delivered care, using outcome indicators explicitly, part of internal policy with concrete objectives? *(multiple answers possible)***

No

Yes, part of the specialism's annual plan

Yes, part of the specialism's multiannual plan

Yes, part of the department's annual plan

Yes, part of the department's multiannual plan

Yes, part of the hospital-wide annual plan

Yes, part of the hospital-wide multiannual plan

Other

**15. When have outcome reports led to improvement initiatives in your hospital in the past 2 years?** Never

If the hospital has significantly less favourable outcomes than the average of other hospitals (national benchmark).

If the reporting leads to clinically relevant insights that can be a starting point for improvements regardless of whether there are statistically significant differences (e.g. a negative trend in the data or outcomes within subpopulations)

If one or more other hospitals have demonstrably more favourable outcomes than average.

Other

**16. How many improvement initiatives have been initiated by monitoring outcomes of care in the past two years?**

[open: numeric field]

**17. Which of the following learning strategies have proved successful in the creation of these improvement initiatives?**

**Best practice**

Never

Rarely

Sometimes

Often

Very often

Not applicable

**Process analysis**

Never

Rarely

Sometimes

Often

Very often

Not applicable

**File analysis of patients**

Never

Rarely

Sometimes

Often

Very often

Not applicable

**Scientific literature**

Never

Rarely

Sometimes

Often

Very often

Not applicable

**Guidelines studied and implemented more rigorously**

Never

Rarely

Sometimes

Often

Very often

Not applicable

**Initiatives based on clinical experience**

Never

Rarely

Sometimes

Often

Very often

Not applicable

**Structural learning environment with other hospitals**

Never

Rarely

Sometimes

Often

Very often

Not applicable

**Consultation of external experts**

Never

Rarely

Sometimes

Often

Very often

Not applicable

**Internal peer sharing of knowledge, experience or techniques**

Never

Rarely

Sometimes

Often

Very often

Not applicable

**18. To what extent is the implementation of improvement initiatives monitored?**

[on a scale of 0-10; 0 = ‘never’, 10 = ‘always’]

**19. To what extent is the effect of improvement initiatives monitored?**

[on a scale of 0-10; 0 = ‘never’, 10 = ‘always’]

**20. In your opinion, how important is improving quality of care based on outcome indicators considered within your specialism?**

[on a scale of 0-10; 0 = ‘very unimportant’, 10 = ‘very important’]

**21. In your opinion, how high is the trust within your specialism to talk openly about aggregated outcomes?**

[on a scale of 0-10; 0 = ‘very low trust’, 10 = ‘very high trust]

**22. In your opinion, how high is the trust within your specialism to speak openly about outcome by operator?**

[on a scale of 0-10; 0 = ‘very low trust, 10 = ‘very high trust]

**23. In your opinion, how high is the trust between specialisms to talk openly about aggregated outcomes?**

[on a scale of 0-10; 0 = ‘very low trust, 10 = ‘very high trust]

**24. Different levels within an organisation are shown below. Please rank the levels in order of the extent to which a leadership role is taken to achieve an improvement cycle based on outcome indicators?**

Physicians

Medical society management

Hospital management

Nurses

Quality department

Board of directors

Staff board

**25. Is there another level within the organisation which takes a leading role to realise an improvement cycle based on outcome indicators?**

Yes

No

**26. If yes, which level?**

Quality department

Internal research committee

Research & Development

Infection prevention

Hospital hygiene department

Supporting services (such as pharmacy)

Other

**27. Is there an explicit (non-)medical person with the responsibility for quality of care for patients?**

No

Yes, the medical manager

Yes, but not the medical manager

Yes, not generic, but different specialists for different conditions

Other

**28. What percentage of data from the quality registration is recorded during regular care in the EHR?** [open: numeric field]

**29. How does the data get submitted to the quality registry?**

Manual input into quality registration application (retyping)

Upload of template filled partly manually and partly by extraction from EHR

Upload of template filled by (almost) full extraction from EHR

Upload of template filled by (almost) full extraction from PDMS

Direct link between EHR and quality registration

Other

**30. How is the data checked for quality before delivery?**

Not

By a data manager

By a physician

Other

**31. How high is your trust in the quality of data from your own hospital from the quality registration?**

[on a scale of 0-10; 0 = ‘very low trust, 10 = ‘very high trust]

**32. How high is your trust in the quality of data from other hospitals from quality registration?**

[on a scale of 0-10; 0 = ‘very low trust, 10 = ‘very high trust]

**33. How often are data uploaded to the quality registry?**

Not

Less than once a year

Once a year

Between once a year and quarterly

Quarterly

Between quarterly and monthly

Monthly

More often than monthly

Unknown

**34. Which of the following officer(s) are provided with time to implement an improvement cycle aimed at improving outcomes of care?**

Medical specialists

Quality managers

Internal consultants

Medical managers

Specialism' managers

Statistician

Researcher

Departmental manager

Not applicable

Other

**35. In total, how many hours per week do these individuals have available of this?**

[open: numeric field]

Not applicable

**36. How many doctors are there in the hospital with clear expertise and affinity for data management and data analytics?**

[open: numeric field]

Not applicable
