# Supplementary material for: The use of outcome data from quality registries to learn and improve; a Dutch nationwide quantitative analysis in five disease areas
Source: BMC Health Serv Res. 2024 Oct 29;24:1296. doi: 10.1186/s12913-024-11760-z (PMC11520663; doi:10.1186/s12913-024-11760-z)
Supplement: Supplementary file 2 — Supplementary Material 2. [file 12913_2024_11760_MOESM2_ESM.docx]

**Supplementary Table 1 - Results per quality registration for each element of the HOME model.**

*Supplementary Table 1. Results per quality registration for each element of the HOME model.*

| **Question** | **Answer options** | **Cardiology** | **Cardiothoracic surgery** | **Intensive care** | **Nephrology** | **Orthopeadic surgery** | **Total** |
| --- | --- | --- | --- | --- | --- | --- | --- |
| **Outcome-based improvement cycle** |  | **N = 20** | **N = 12** | **N = 35** | **N = 30** | **N = 27** | **N = 124** |
| **1. Monitoring of outcomes** |  |  |  |  |  |  |  |
| How frequent are health outcomes measured and discussed within the department? | Not | 0 (0%) | 0 (0%) | 0 (0%) | 0 (0%) | 0 (0%) | 0 (0%) |
|  | Less than once a year | 0 (0%) | 0 (0%) | 0 (0%) | 0 (0%) | 0 (0%) | 0 (0%) |
|  | Once a year | 7 (35%) | 1 (8.3%) | 7 (20%) | 1 (3.3%) | 3 (11.1%) | 19 (15.3%) |
|  | Between once a year and quarterly | 5 (25%) | 3 (25%) | 16 (45.7%) | 8 (26.6%) | 9 (33.3%) | 41 (33.1%) |
|  | Quarterly | 4 (20%) | 4 (33.3%) | 7 (20%) | 19 (63.3%) | 6 (22.2%) | 40 (32.3%) |
|  | Between quarterly and monthly | 2 (10%) | 1 (8.3%) | 4 (11.4%) | 1 (3.3%) | 5 (18.5%) | 13 (10.5%) |
|  | Monthly | 1 (5%) | 3 (25%) | 1 (2.9%) | 1 (3.3%) | 2 (7.4%) | 8 (6.45%) |
|  | More often than monthly | 1 (5%) | 0 (0%) | 0 (0%) | 0 (0%) | 2 (7.4%) | 3 (2.4%) |
|  | Unknown | 0 (0%) | 0 (0%) | 0 (0%) | 0 (0%) | 0 (0%) | 0 (0%) |
| How frequent are health outcomes measured and discussed in a multidisciplinary setting? | Not | 9 (45%) | 2 (16.6%) | 14 (40%) | 2 (6.6%) | 5 (18.5%) | 32 (25.8%) |
|  | Less than once a year | 0 (0%) | 0 (0%) | 4 (11.4%) | 1 (3.3%) | 1 (3.7%) | 6 (4.8%) |
|  | Once a year | 5 (25%) | 0 (0%) | 8 (22.9%) | 2 (6.6%) | 6 (22.2%) | 21 (16.9%) |
|  | Between once a year and quarterly | 3 (15%) | 3 (25%) | 7 (20%) | 11 (36.6%) | 6 (22.2%) | 30 (24.2%) |
|  | Quarterly | 2 (10%) | 3 (25%) | 1 (2.9%) | 11 (36.6%) | 3 (11.1%) | 20 (16.1%) |
|  | Between quarterly and monthly | 0 (0%) | 0 (0%) | 0 (0%) | 1 (3.3%) | 4 (14.8%) | 5 (4.0%) |
|  | Monthly | 0 (0%) | 2 (16.6%) | 0 (0%) | 0 (0%) | 2 (7.4%) | 4 (3.2%) |
|  | More often than monthly | 1 (5%) | 2 (16.6%) | 0 (0%) | 2 (6.6%) | 0 (0%) | 5 (4.0%) |
|  | Unknown | 0 (0%) | 0 (0%) | 1 (2.9%) | 0 (0%) | 0 (0%) | 1 (0.8%) |
| Which other parties within your hospital, apart from the multidisciplinary meetings or department meetings, are involved in the measurement and discussion of outcome measures? *Multiple answers possible* | None | 6 (24%) | 4 (22.2%) | 10 (18.86%) | 4 (4.76%) | 1 (1.69%) | 25 (10.5%) |
|  | Patients | 0 (0%) | 0 (0%) | 0 (0%) | 9 (10.7%) | 5 (8.47%) | 14 (5.9%) |
|  | Hospital management | 14 (56%) | 7 (38.9%) | 19 (35.8%) | 26 (31%) | 25 (42.4%) | 91 (38.1%) |
|  | Staff board | 0 (0%) | 0 (0%) | 7 (13.2%) | 6 (7.1%) | 9 (15.3%) | 22 (9.2%) |
|  | Board of directors | 3 (12%) | 6 (33.3%) | 12 (22.6%) | 19 (22.6%) | 14 (23.7%) | 54 (22.6%) |
|  | Other | 2 (8%) | 1 (5.6%) | 5 (9.4%) | 20 (23.8%) | 5 (8.47%) | 33 (13.8%) |
| How many physicians that are directly involved in the treatment of patients are involved in the discussion of outcome measures? | 1 to 5 | 4 (20%) | 2 (16.6%) | 7 (20%) | 10 (33.3%) | 9 (33.3%) | 32 (25.8%) |
|  | 6 to 10 | 6 (30%) | 3 (25%) | 8 (22.9%) | 12 (40%) | 13 (48.1%) | 42 (33.9%) |
|  | 11 to 20 | 5 (25%) | 2 (16.6%) | 9 (25.7%) | 5 (16.6%) | 4 (14.8%) | 25 (20.2%) |
|  | 21 to 50 | 4 (20%) | 4 (33.3%) | 9 (25.7%) | 3 (10%) | 1 (3.7%) | 21 (16.9%) |
|  | 51 to 99 | 1 (5%) | 1 (8.3%) | 0 (0%) | 0 (0%) | 0 (0%) | 2 (1.6%) |
|  | More than 100 | 0 (0%) | 0 (0%) | 2 (5.7%) | 0 (0%) | 0 (0%) | 2 (1.6%) |
| How many physicians take active knowledge of health outcomes of your hospital? | 0% | 0 (0%) | 0 (0%) | 3 (8.6%) | 1 (3.3%) | 0 (0%) | 4 (3.2%) |
|  | 1-25% | 4 (20%) | 2 (16.6%) | 11 (31.4%) | 4 (13.3%) | 10 (37.0%) | 31 (25%) |
|  | 26-50% | 3 (15%) | 1 (8.3%) | 5 (14.3%) | 6 (20%) | 1 (3.7%) | 16 (12.9%) |
|  | 51-75% | 1 (5%) | 1 (8.3%) | 1 (2.9%) | 4 (13.3%) | 0 (0%) | 7 (5.6%) |
|  | 76-99% | 3 (15%) | 4 (33.3%) | 3 (8.6%) | 2 (6.6%) | 1 (3.7%) | 13 (10.5%) |
|  | 100% | 9 (45%) | 3 (25%) | 13 (37.1%) | 13 (43.3%) | 15 (55.6%) | 53 (42.7%) |
| How frequent are (digital) dashboards, in which health outcomes are displayed, used to discuss health outcomes within your speciality? | **Self developed dashboards** |  |  |  |  |  |  |
|  | Never | 8 (40%) | 3 (25%) | 20 (57.1%) | 12 (40%) | 4 (14.8%) | 47 (37.9%) |
|  | Rarely | 4 (20%) | 0 (0%) | 5 (14.3%) | 0 (0%) | 3 (11.1%) | 12 (9.7%) |
|  | Sometimes | 3 (15%) | 3 (25%) | 7 (20%) | 6 (20%) | 9 (33.3%) | 28 (22.6%) |
|  | Often | 3 (15%) | 4 (33.3%) | 2 (5.7%) | 6 (20%) | 8 (29.6%) | 23 (18.5%) |
|  | Very often | 2 (10%) | 2 (16.6%) | 1 (2.9%) | 6 (20%) | 3 (11.1%) | 14 (11.3%) |
|  | **CQR dashboards** |  |  |  |  |  |  |
|  | Never | 1 (5%) | 0 (0%) | 1 (2.9%) | 0 (0%) | 0 (0%) | 2 (1.6%) |
|  | Rarely | 4 (20%) | 2 (16.6%) | 7 (20%) | 3 (10%) | 4 (14.8%) | 20 (16.1%) |
|  | Sometimes | 11 (55%) | 6 (50%) | 16 (45.7%) | 15 (50%) | 14 (51.9%) | 62 (50%) |
|  | Often | 4 (20%) | 4 (33.3%) | 8 (22.9%) | 8 (26.6%) | 8 (29.6%) | 32 (25.8%) |
|  | Very often | 0 (0%) | 0 (0%) | 3 (8.6%) | 4 (13.3%) | 1 (3.7%) | 8 (6.45%) |
| Does your apartment use additional health outcomes besides information from the quality registry? | Yes | 12 (60%) | 10 (83.3%) | 21 (60%) | 27 (90%) | 25 (92.6%) | 95 (76.6%) |
|  | No | 7 (35%) | 1 (8.3%) | 12 (34.3%) | 3 (10%) | 2 (7.4%) | 25 (20.2%) |
|  | Don't know/other | 1 (5%) | 1 (8.3%) | 2 (5.7%) | 0 (0%) | 0 (0%) | 4 (3.2%) |
| **2. Identification of improvement potential** |  |  |  |  |  |  |  |
| How frequent are the following types of analyses performed to identify improvement potential? | **Uncorrected health outcomes of your hospital** |  |  |  |  |  |  |
|  | Never | 1 (5%) | 0 (0%) | 3 (8.6%) | 0 (0%) | 1 (3.7%) | 5 (4.0%) |
|  | Rarely | 3 (15%) | 0 (0%) | 5 (14.3%) | 2 (6.6%) | 1 (3.7%) | 11 (8.9%) |
|  | Sometimes | 6 (30%) | 2 (16.6%) | 16 (45.7%) | 6 (20%) | 11 (40.7%) | 41 (33.1%) |
|  | Often | 6 (30%) | 7 (58.3%) | 11 (31.4%) | 20 (66.6%) | 13 (48.1%) | 57 (46%) |
|  | Very often | 4 (20%) | 3 (25%) | 0 (0%) | 2 (6.6%) | 1 (3.7%) | 10 (8.1%) |
|  | **Risk-corrected health outcomes of your hospital** |  |  |  |  |  |  |
|  | Never | 3 (15%) | 0 (0%) | 6 (17.1%) | 2 (6.6%) | 4 (14.8%) | 15 (12.1%) |
|  | Rarely | 3 (15%) | 2 (16.6%) | 5 (14.3%) | 3 (10%) | 5 (18.5%) | 18 (14.5%) |
|  | Sometimes | 5 (25%) | 4 (33.3%) | 13 (37.1%) | 7 (23.3%) | 6 (22.2%) | 35 (28.2%) |
|  | Often | 6 (30%) | 4 (33.3%) | 10 (28.6%) | 17 (56.6%) | 12 (44.4%) | 49 (39.5%) |
|  | Very often | 3 (15%) | 2 (16.6%) | 1 (2.9%) | 1 (3.3%) | 0 (0%) | 7 (5.6%) |
|  | **Uncorrected health outcomes compared to other hospitals** |  |  |  |  |  |  |
|  | Never | 2 (10%) | 0 (0%) | 5 (14.3%) | 3 (10%) | 5 (18.5%) | 15 (12.1%) |
|  | Rarely | 4 (20%) | 4 (33.3%) | 7 (20%) | 5 (16.6%) | 5 (18.5%) | 25 (20.2%) |
|  | Sometimes | 9 (45%) | 4 (33.3%) | 16 (45.7%) | 13 (43.3%) | 12 (44.4%) | 54 (43.5%) |
|  | Often | 2 (10%) | 3 (25%) | 7 (20%) | 7 (23.3%) | 4 (14.8%) | 23 (18.5%) |
|  | Very often | 3 (15%) | 1 (8.3%) | 0 (0%) | 2 (6.6%) | 1 (3.7%) | 7 (5.6%) |
|  | **Risk-corrected health outcomes compared to other hospitals** |  |  |  |  |  |  |
|  | Never | 3 (15%) | 0 (0%) | 5 (14.3%) | 4 (13.3%) | 7 (25.9%) | 19 (15.3%) |
|  | Rarely | 3 (15%) | 3 (25%) | 7 (20%) | 5 (16.6%) | 8 (29.6%) | 26 (21%) |
|  | Sometimes | 8 (40%) | 3 (25%) | 15 (42.9%) | 11 (36.6%) | 9 (33.3%) | 46 (37.1%) |
|  | Often | 4 (20%) | 5 (41.6%) | 8 (22.9%) | 9 (30%) | 3 (11.1%) | 29 (23.4%) |
|  | Very often | 2 (10%) | 1 (8.3%) | 0 (0%) | 1 (3.3%) | 0 (0%) | 4 (3.2%) |
|  | **Inter-doctor variation in uncorrected health outcomes** |  |  |  |  |  |  |
|  | Never | 10 (50%) | 4 (33.3%) | n/a | 12 (40%) | 5 (18.5%) | 31 (25%) |
|  | Rarely | 7 (35%) | 3 (25%) | n/a | 7 (23.3%) | 4 (14.8%) | 21 (16.9%) |
|  | Sometimes | 1 (5%) | 2 (16.6%) | n/a | 5 (16.6%) | 14 (51.9%) | 22 (17.7%) |
|  | Often | 1 (5%) | 1 (8.3%) | n/a | 4 (13.3%) | 4 (14.8%) | 10 (8.1%) |
|  | Very often | 1 (5%) | 2 (16.6%) | n/a | 2 (6.6%) | 0 (0%) | 5 (4.0%) |
|  | **Inter-doctor variation in risk-corrected health outcomes** |  |  |  |  |  |  |
|  | Never | 12 (60%) | 6 (50%) | n/a | 14 (46.6%) | 9 (33.3%) | 41 (33.1%) |
|  | Rarely | 7 (35%) | 1 (8.3%) | n/a | 9 (30%) | 7 (25.9%) | 24 (19.4%) |
|  | Sometimes | 0 (0%) | 2 (16.6%) | n/a | 3 (10%) | 9 (33.3%) | 14 (11.3%) |
|  | Often | 1 (5%) | 2 (16.6%) | n/a | 2 (6.6%) | 2 (7.4%) | 7 (5.6%) |
|  | Very often | 0 (0%) | 1 (8.3%) | n/a | 2 (6.6%) | 0 (0%) | 3 (2.4%) |
| Have targets been set for outcomes measures in the last two years within your department? | No | 7 (35%) | 4 (33.3%) | 13 (37.1%) | 5 (16.6%) | 4 (14.8%) | 33 (26.6%) |
|  | Yes, for one or some health outcomes | 10 (50%) | 4 (33.3%) | 17 (48.6%) | 12 (40%) | 12 (44.4%) | 55 (44.4%) |
|  | Yes, for one or some health outcomes and for at least one of the medical conditions for which outcomes are available | 2 (10%) | 3 (25%) | 0 (0%) | 4 (13.3%) | 7 (25.9%) | 16 (12.9%) |
|  | Yes, for all health outcomes | 0 (0%) | 0 (0%) | 3 (8.6%) | 6 (20%) | 3 (11.1%) | 12 (9.7%) |
|  | Other | 1 (5%) | 1 (8.3%) | 2 (5.7%) | 2 (6.6%) | 1 (3.7%) | 7 (5.6%) |
| When have outcome reports led to improvement initiatives in your hospital in the past 2 years? | Never | 3 (15%) | 2 (16.6%) | 6 (17.1%) | 0 (0%) | 1 (3.7%) | 12 (9.7%) |
|  | If the hospital has significantly less favourable outcomes than the average of other hospitals (national benchmark). | 6 (30%) | 5 (41.6%) | 11 (31.4%) | 14 (46.6%) | 9 (33.3%) | 45 (36.3%) |
|  | If the reporting leads to clinically relevant insights that can be a starting point for improvements regardless of whether there are statistically significant differences (e.g. a negative trend in the data or outcomes within subpopulations) | 13 (65%) | 9 (75%) | 24 (68.6%) | 23 (76.6%) | 17 (63%) | 86 (69.4%) |
|  | If one or more other hospitals have demonstrably more favourable outcomes than average. | 1 (5%) | 3 (25%) | 2 (5.7%) | 6 (20%) | 1 (3.7%) | 13 (10.5%) |
|  | Other | 1 (5%) | 0 (0%) | 2 (5.7%) | 1 (3.3%) | 1 (3.7%) | 5 (4.0%) |
| **3. Selection of improvement initiatives** |  |  |  |  |  |  |  |
| For how many outcome indicators have additional analyses been carried out in the past two years at the initiative or request of your specialism with the aim of better interpreting results and possible arriving at improvement initiatives? | None | 1 (5%) | 2 (16.6%) | 12 (34.3%) | 4 (13.3%) | 3 (11.1%) | 22 (17.7%) |
|  | 1 | 7 (35%) | 0 (0%) | 3 (8.6%) | 1 (3.3%) | 0 (0%) | 11 (8.9%) |
|  | 2 to 4 | 9 (45%) | 8 (66.6%) | 11 (31.4%) | 19 (63.3%) | 20 (74.1%) | 67 (54.0%) |
|  | 5 to 10 | 2 (10%) | 2 (16.6%) | 7 (20%) | 5 (16.6%) | 1 (3.7%) | 17 (13.7%) |
|  | More than 10 | 1 (5%) | 0 (0%) | 2 (5.7%) | 1 (3.3%) | 3 (11.1%) | 7 (5.6%) |
| How many improvement initiatives have been initiated by monitoring outcomes of care in the past two years? | None | 4 (20%) | 2 (16.6%) | 7 (20%) | 1 (3.3%) | 1 (3.7%) | 15 (12.1%) |
|  | 1 | 6 (30%) | 1 (8.3%) | 7 (20%) | 1 (3.3%) | 3 (11.1%) | 18 (14.5%) |
|  | 2 to 4 | 8 (40%) | 6 (50%) | 20 (57.1%) | 20 (66.6%) | 15 (55.6%) | 69 (55.6%) |
|  | 5 to 10 | 2 (10%) | 3 (25%) | 0 (0%) | 7 (23.3%) | 7 (25.9%) | 19 (15.3%) |
|  | More than 10 | 0 (0%) | 0 (0%) | 1 (2.9%) | 1 (3.3%) | 1 (3.7%) | 3 (2.4%) |
| Which of the following learning strategies have proved successful in the creation of these improvement initiatives? | **Best practice** |  |  |  |  |  |  |
|  | Never | 3 (15%) | 0 (0%) | 5 (14.3%) | 0 (0%) | 1 (3.7%) | 9 (7.3%) |
|  | Rarely | 5 (25%) | 3 (25%) | 6 (17.1%) | 6 (20%) | 4 (14.8%) | 24 (19.4%) |
|  | Sometimes | 8 (40%) | 5 (41.6%) | 13 (37.1%) | 13 (43.3%) | 10 (37.0%) | 49 (39.5%) |
|  | Often | 2 (10%) | 3 (25%) | 7 (20%) | 7 (23.3%) | 7 (25.9%) | 26 (21%) |
|  | Very often | 0 (0%) | 0 (0%) | 0 (0%) | 2 (6.6%) | 3 (11.1%) | 5 (4.0%) |
|  | Not applicable | 2 (10%) | 1 (8.3%) | 4 (11.4%) | 2 (6.6%) | 2 (7.4%) | 11 (8.9%) |
|  | **Process analysis** |  |  |  |  |  |  |
|  | Never | 6 (30%) | 3 (25%) | 7 (20%) | 4 (13.3%) | 2 (7.4%) | 22 (17.7%) |
|  | Rarely | 1 (5%) | 0 (0%) | 9 (25.7%) | 6 (20%) | 3 (11.1%) | 19 (15.3%) |
|  | Sometimes | 6 (30%) | 5 (41.6%) | 4 (11.4%) | 5 (16.6%) | 8 (29.6%) | 28 (22.6%) |
|  | Often | 3 (15%) | 1 (8.3%) | 6 (17.1%) | 8 (26.6%) | 7 (25.9%) | 25 (20.2%) |
|  | Very often | 0 (0%) | 1 (8.3%) | 1 (2.9%) | 4 (13.3%) | 3 (11.1%) | 9 (7.3%) |
|  | Not applicable | 4 (20%) | 2 (16.6%) | 8 (22.9%) | 3 (10%) | 4 (14.8%) | 21 (16.9%) |
|  | **File analysis of patients** |  |  |  |  |  |  |
|  | Never | 1 (5%) | 0 (0%) | 4 (11.4%) | 0 (0%) | 2 (7.4%) | 7 (5.6%) |
|  | Rarely | 0 (0%) | 1 (8.3%) | 2 (5.7%) | 2 (6.6%) | 1 (3.7%) | 6 (4.8%) |
|  | Sometimes | 8 (40%) | 2 (16.6%) | 7 (20%) | 12 (40%) | 6 (22.2%) | 35 (28.2%) |
|  | Often | 7 (35%) | 6 (50%) | 17 (48.6%) | 13 (43.3%) | 11 (40.7%) | 54 (43.5%) |
|  | Very often | 3 (15%) | 2 (16.6%) | 4 (11.4%) | 2 (6.6%) | 5 (18.5%) | 16 (12.9%) |
|  | Not applicable | 1 (5%) | 1 (8.3%) | 1 (2.9%) | 1 (3.3%) | 2 (7.4%) | 6 (4.8%) |
|  | **Scientific literature** |  |  |  |  |  |  |
|  | Never | 1 (5%) | 0 (0%) | 1 (2.9%) | 0 (0%) | 1 (3.7%) | 3 (2.4%) |
|  | Rarely | 2 (10%) | 2 (16.6%) | 2 (5.7%) | 0 (0%) | 1 (3.7%) | 7 (5.6%) |
|  | Sometimes | 5 (25%) | 1 (8.3%) | 9 (25.7%) | 13 (43.3%) | 8 (29.6%) | 36 (29.0%) |
|  | Often | 7 (35%) | 5 (41.6%) | 18 (51.4%) | 11 (36.6%) | 10 (37.0%) | 51 (41.1%) |
|  | Very often | 4 (20%) | 3 (25%) | 5 (14.3%) | 6 (20%) | 7 (25.9%) | 25 (20.2%) |
|  | Not applicable | 1 (5%) | 1 (8.3%) | 0 (0%) | 0 (0%) | 0 (0%) | 2 (1.6%) |
|  | **Guidelines studied and implemented more rigorously** |  |  |  |  |  |  |
|  | Never | 2 (10%) | 0 (0%) | 2 (5.7%) | 0 (0%) | 0 (0%) | 4 (3.2%) |
|  | Rarely | 1 (5%) | 4 (33.3%) | 3 (8.6%) | 0 (0%) | 0 (0%) | 8 (6.45%) |
|  | Sometimes | 8 (40%) | 1 (8.3%) | 11 (31.4%) | 11 (36.6%) | 12 (44.4%) | 43 (34.7%) |
|  | Often | 5 (25%) | 4 (33.3%) | 15 (42.9%) | 15 (50%) | 11 (40.7%) | 50 (40.3%) |
|  | Very often | 3 (15%) | 2 (16.6%) | 4 (11.4%) | 4 (13.3%) | 4 (14.8%) | 17 (13.7%) |
|  | Not applicable | 1 (5%) | 1 (8.3%) | 0 (0%) | 0 (0%) | 0 (0%) | 2 (1.6%) |
|  | **Initiatives based on clinical experience** |  |  |  |  |  |  |
|  | Never | 2 (10%) | 0 (0%) | 1 (2.9%) | 0 (0%) | 1 (3.7%) | 4 (3.2%) |
|  | Rarely | 1 (5%) | 3 (25%) | 6 (17.1%) | 0 (0%) | 6 (22.2%) | 16 (12.9%) |
|  | Sometimes | 7 (35%) | 1 (8.3%) | 17 (48.6%) | 11 (36.6%) | 6 (22.2%) | 42 (33.9%) |
|  | Often | 6 (30%) | 6 (50%) | 11 (31.4%) | 13 (43.3%) | 10 (37.0%) | 46 (37.1%) |
|  | Very often | 3 (15%) | 1 (8.3%) | 0 (0%) | 5 (16.6%) | 3 (11.1%) | 12 (9.7%) |
|  | Not applicable | 1 (5%) | 1 (8.3%) | 0 (0%) | 1 (3.3%) | 1 (3.7%) | 4 (3.2%) |
|  | **Structural learning environment with other hospitals** |  |  |  |  |  |  |
|  | Never | 3 (15%) | 1 (8.3%) | 9 (25.7%) | 2 (6.6%) | 4 (14.8%) | 19 (15.3%) |
|  | Rarely | 2 (10%) | 4 (33.3%) | 5 (14.3%) | 8 (26.6%) | 5 (18.5%) | 24 (19.4%) |
|  | Sometimes | 8 (40%) | 4 (33.3%) | 14 (40%) | 9 (30%) | 7 (25.9%) | 42 (33.9%) |
|  | Often | 5 (25%) | 1 (8.3%) | 4 (11.4%) | 2 (6.6%) | 6 (22.2%) | 18 (14.5%) |
|  | Very often | 0 (0%) | 1 (8.3%) | 1 (2.9%) | 5 (16.6%) | 2 (7.4%) | 9 (7.3%) |
|  | Not applicable | 2 (10%) | 1 (8.3%) | 2 (5.7%) | 4 (13.3%) | 3 (11.1%) | 12 (9.7%) |
|  | **Consultation of external experts** |  |  |  |  |  |  |
|  | Never | 7 (35%) | 4 (33.3%) | 12 (34.3%) | 4 (13.3%) | 8 (29.6%) | 35 (28.2%) |
|  | Rarely | 3 (15%) | 3 (25%) | 8 (22.9%) | 7 (23.3%) | 8 (29.6%) | 29 (23.4%) |
|  | Sometimes | 4 (20%) | 3 (25%) | 9 (25.7%) | 10 (33.3%) | 8 (29.6%) | 34 (27.4%) |
|  | Often | 1 (5%) | 1 (8.3%) | 2 (5.7%) | 6 (20%) | 0 (0%) | 10 (8.1%) |
|  | Very often | 0 (0%) | 0 (0%) | 0 (0%) | 1 (3.3%) | 0 (0%) | 1 (0.8%) |
|  | Not applicable | 5 (25%) | 1 (8.3%) | 4 (11.4%) | 2 (6.6%) | 3 (11.1%) | 15 (12.1%) |
|  | **Internal peer sharing of knowledge, experience or techniques** |  |  |  |  |  |  |
|  | Never | 0 (0%) | 0 (0%) | 5 (14.3%) | 0 (0%) | 1 (3.7%) | 6 (4.8%) |
|  | Rarely | 2 (10%) | 3 (25%) | 1 (2.9%) | 2 (6.6%) | 1 (3.7%) | 9 (7.3%) |
|  | Sometimes | 6 (30%) | 3 (25%) | 10 (28.6%) | 3 (10%) | 6 (22.2%) | 28 (22.6%) |
|  | Often | 5 (25%) | 3 (25%) | 13 (37.1%) | 16 (53.3%) | 12 (44.4%) | 49 (39.5%) |
|  | Very often | 5 (25%) | 2 (16.6%) | 5 (14.3%) | 9 (30%) | 7 (25.9%) | 28 (22.6%) |
|  | Not applicable | 2 (10%) | 1 (8.3%) | 1 (2.9%) | 0 (0%) | 0 (0%) | 4 (3.2%) |
| **4. Implementation of improvement initiatives** |  |  |  |  |  |  |  |
| To what extent is the implementation of improvement initiatives monitored? (on a scale of 0-10) | 0 | 3 (15%) | 2 (16.6%) | 1 (2.9%) | 0 (0%) | 0 (0%) | 6 (4.8%) |
|  | 1 | 0 (0%) | 0 (0%) | 0 (0%) | 0 (0%) | 0 (0%) | 0 (0%) |
|  | 2 | 1 (5%) | 0 (0%) | 2 (5.7%) | 0 (0%) | 1 (3.7%) | 4 (3.2%) |
|  | 3 | 1 (5%) | 1 (8.3%) | 2 (5.7%) | 0 (0%) | 1 (3.7%) | 5 (4.0%) |
|  | 4 | 0 (0%) | 0 (0%) | 3 (8.6%) | 2 (6.6%) | 1 (3.7%) | 6 (4.8%) |
|  | 5 | 5 (25%) | 1 (8.3%) | 5 (14.3%) | 1 (3.3%) | 1 (3.7%) | 13 (10.5%) |
|  | 6 | 4 (20%) | 2 (16.6%) | 6 (17.1%) | 7 (23.3%) | 5 (18.5%) | 24 (19.4%) |
|  | 7 | 3 (15%) | 2 (16.6%) | 4 (11.4%) | 9 (30%) | 10 (37.0%) | 28 (22.6%) |
|  | 8 | 3 (15%) | 2 (16.6%) | 6 (17.1%) | 6 (20%) | 5 (18.5%) | 22 (17.7%) |
|  | 9 | 0 (0%) | 1 (8.3%) | 2 (5.7%) | 3 (10%) | 2 (7.4%) | 8 (6.45%) |
|  | 10 | 0 (0%) | 1 (8.3%) | 4 (11.4%) | 2 (6.6%) | 1 (3.7%) | 8 (6.45%) |
| To what extent is the effect of improvement initiatives monitored? (on a scale of 0-10) | 0 | 3 (15%) | 2 (16.6%) | 1 (2.9%) | 0 (0%) | 0 (0%) | 6 (4.8%) |
|  | 1 | 0 (0%) | 0 (0%) | 1 (2.9%) | 0 (0%) | 0 (0%) | 1 (0.8%) |
|  | 2 | 0 (0%) | 0 (0%) | 2 (5.7%) | 0 (0%) | 1 (3.7%) | 3 (2.4%) |
|  | 3 | 3 (15%) | 0 (0%) | 3 (8.6%) | 1 (3.3%) | 1 (3.7%) | 8 (6.45%) |
|  | 4 | 0 (0%) | 0 (0%) | 1 (2.9%) | 0 (0%) | 0 (0%) | 1 (0.8%) |
|  | 5 | 2 (10%) | 0 (0%) | 6 (17.1%) | 2 (6.6%) | 1 (3.7%) | 11 (8.9%) |
|  | 6 | 3 (15%) | 1 (8.3%) | 4 (11.4%) | 7 (23.3%) | 5 (18.5%) | 20 (16.1%) |
|  | 7 | 6 (30%) | 3 (25%) | 7 (20%) | 9 (30%) | 4 (14.8%) | 29 (23.4%) |
|  | 8 | 2 (10%) | 3 (25%) | 5 (14.3%) | 6 (20%) | 9 (33.3%) | 25 (20.2%) |
|  | 9 | 1 (5%) | 1 (8.3%) | 2 (5.7%) | 3 (10%) | 4 (14.8%) | 11 (8.9%) |
|  | 10 | 0 (0%) | 2 (16.6%) | 3 (8.6%) | 2 (6.6%) | 2 (7.4%) | 9 (7.3%) |

| **Organizational context** |  | **Cardiology (PCI)** | **Cardiothoracic surgery** | **Intensive care** | **Nephrology** | **Orthopeadic surgery** | **Total** |
| --- | --- | --- | --- | --- | --- | --- | --- |
| **1. Strategy** |  | **N = 20** | **N = 12** | **N = 35** | **N = 30** | **N = 27** | **N = 124** |
| Is the measuring and improving of outcomes of the delivered care, using outcome indicators explicitly, part of internal policy with concrete objectives? *(multiple answers possible)* | No | 5 (20%) | 1 (5.6%) | 3 (5.6%) | 1 (1.2%) | 1 (1.69%) | 11 (4.6%) |
|  | Yes, part of the specialism's annual plan | 6 (24%) | 5 (27.8%) | 24 (45.3%) | 6 (7.1%) | 14 (23.7%) | 55 (23.0%) |
|  | Yes, part of the specialism's multiannual plan | 9 (36%) | 0 (0%) | 15 (28.3%) | 5 (5.95%) | 15 (25.4%) | 44 (18.4%) |
|  | Yes, part of the department's annual plan | 5 (20%) | 7 (38.88%) | 1 (1.88%) | 25 (29.76%) | 7 (11.86%) | 45 (18.8%) |
|  | Yes, part of the department's multiannual plan | 3 (12%) | 5 (27.8%) | 2 (3.8%) | 10 (11.9%) | 5 (8.47%) | 25 (10.5%) |
|  | Yes, part of the hospital-wide annual plan | 5 (20%) | 1 (5.6%) | 5 (9.4%) | 8 (9.52%) | 8 (13.6%) | 27 (11.3%) |
|  | Yes, part of the hospital-wide multiannual plan | 6 (24%) | 3 (16.6%) | 9 (16.98%) | 6 (7.1%) | 6 (10.2%) | 30 (12.6%) |
|  | Other | 0 (0%) | 0 (0%) | 1 (1.88%) | 2 (2.4%) | 2 (3.4%) | 5 (2.1%) |
| **2. Governance** |  |  |  |  |  |  |  |
| Which parties within the entire chain of care delivery, besides the parties involved in your own hospital, are involved in the measurement and discussion of health outcomes? *Multiple answers possible* | None | 8 (36.4%) | 5 (38.5%) | 10 (27.8%) | 14 (42.4%) | 11 (33.3%) | 48 (35.0%) |
|  | Referring hospitals | 5 (22.7%) | 7 (53.8%) | 1 (2.8%) | 1 (3.0%) | 1 (3.0%) | 15 (10.9%) |
|  | Network or collaboration with other hospitals | 8 (36.4%) | 1 (7.69%) | 23 (63.9%) | 13 (39.4%) | 10 (30.3%) | 55 (40.1%) |
|  | General practitioners | 1 (4.5%) | 0 (0%) | 0 (0%) | 1 (3.0%) | 5 (15.15%) | 7 (5.1%) |
|  | Nursery homes | 0 (0%) | 0 (0%) | 0 (0%) | 0 (0%) | 2 (6.1%) | 2 (1.5%) |
|  | Revalidation | 0 (0%) | 0 (0%) | 1 (2.8%) | 0 (0%) | 1 (3.0%) | 2 (1.5%) |
|  | Domestic care | 0 (0%) | 0 (0%) | 0 (0%) | 0 (0%) | 0 (0%) | 0 (0%) |
|  | Mental health services | 0 (0%) | 0 (0%) | 0 (0%) | 0 (0%) | 0 (0%) | 0 (0%) |
|  | Health insurer | 0 (0%) | 0 (0%) | 1 (2.8%) | 1 (3.0%) | 2 (6.1%) | 4 (2.9%) |
|  | Other | 0 (0%) | 0 (0%) | 0 (0%) | 3 (9.1%) | 1 (3.0%) | 4 (2.9%) |
| **3. Culture** |  |  |  |  |  |  |  |
| In your opinion, how important is improving quality of care based on outcome indicators considered within your specialism? (On a scale of 0-10) | 0 | 0 (0%) | 0 (0%) | 0 (0%) | 0 (0%) | 0 (0%) | 0 (0%) |
|  | 1 | 0 (0%) | 0 (0%) | 0 (0%) | 0 (0%) | 0 (0%) | 0 (0%) |
|  | 2 | 0 (0%) | 0 (0%) | 1 (2.9%) | 0 (0%) | 0 (0%) | 1 (0.8%) |
|  | 3 | 0 (0%) | 0 (0%) | 2 (5.7%) | 0 (0%) | 1 (3.7%) | 3 (2.4%) |
|  | 4 | 2 (10%) | 0 (0%) | 1 (2.9%) | 0 (0%) | 0 (0%) | 3 (2.4%) |
|  | 5 | 2 (10%) | 0 (0%) | 2 (5.7%) | 1 (3.3%) | 0 (0%) | 5 (4.0%) |
|  | 6 | 3 (15%) | 0 (0%) | 4 (11.4%) | 1 (3.3%) | 1 (3.7%) | 9 (7.3%) |
|  | 7 | 4 (20%) | 3 (25%) | 9 (25.7%) | 11 (36.6%) | 7 (25.9%) | 34 (27.4%) |
|  | 8 | 5 (25%) | 3 (25%) | 13 (37.1%) | 11 (36.6%) | 12 (44.4%) | 44 (35.5%) |
|  | 9 | 3 (15%) | 3 (25%) | 1 (2.9%) | 5 (16.6%) | 4 (14.8%) | 16 (12.9%) |
|  | 10 | 1 (5%) | 3 (25%) | 2 (5.7%) | 1 (3.3%) | 2 (7.4%) | 9 (7.3%) |
| In your opinion, how high is the trust within your specialism to talk openly about aggregated outcomes? (On a scale of 0-10) | 0 | 0 (0%) | 0 (0%) | 0 (0%) | 0 (0%) | 0 (0%) | 0 (0%) |
|  | 1 | 0 (0%) | 0 (0%) | 0 (0%) | 0 (0%) | 0 (0%) | 0 (0%) |
|  | 2 | 0 (0%) | 0 (0%) | 0 (0%) | 0 (0%) | 0 (0%) | 0 (0%) |
|  | 3 | 0 (0%) | 0 (0%) | 0 (0%) | 0 (0%) | 1 (3.7%) | 1 (0.8%) |
|  | 4 | 1 (5%) | 0 (0%) | 1 (2.9%) | 0 (0%) | 0 (0%) | 2 (1.6%) |
|  | 5 | 0 (0%) | 0 (0%) | 3 (8.6%) | 0 (0%) | 0 (0%) | 3 (2.4%) |
|  | 6 | 0 (0%) | 0 (0%) | 0 (0%) | 0 (0%) | 3 (11.1%) | 3 (2.4%) |
|  | 7 | 2 (10%) | 2 (16.6%) | 4 (11.4%) | 3 (10%) | 3 (11.1%) | 14 (11.3%) |
|  | 8 | 10 (50%) | 6 (50%) | 9 (25.7%) | 10 (33.3%) | 5 (18.5%) | 40 (32.3%) |
|  | 9 | 6 (30%) | 2 (16.6%) | 9 (25.7%) | 12 (40%) | 9 (33.3%) | 38 (30.6%) |
|  | 10 | 1 (5%) | 2 (16.6%) | 9 (25.7%) | 5 (16.6%) | 6 (22.2%) | 23 (18.5%) |
| In your opinion, how high is the trust within your specialism to speak openly about outcome by operator? (On a scale of 0-10) | 0 | 1 (5%) | 0 (0%) | n.v.t. (%) | 0 (0%) | 0 (0%) | 1 (1.1%) |
|  | 1 | 0 (0%) | 0 (0%) | n.v.t. (%) | 0 (0%) | 0 (0%) | 0 (0%) |
|  | 2 | 0 (0%) | 1 (8.3%) | n.v.t. (%) | 0 (0%) | 1 (3.7%) | 2 (2.24%) |
|  | 3 | 0 (0%) | 0 (0%) | n.v.t. (%) | 0 (0%) | 0 (0%) | 0 (0%) |
|  | 4 | 0 (0%) | 0 (0%) | n.v.t. (%) | 0 (0%) | 0 (0%) | 0 (0%) |
|  | 5 | 2 (10%) | 0 (0%) | n.v.t. (%) | 2 (6.6%) | 1 (3.7%) | 5 (5.6%) |
|  | 6 | 5 (25%) | 0 (0%) | n.v.t. (%) | 1 (3.3%) | 2 (7.4%) | 8 (8.98%) |
|  | 7 | 4 (20%) | 3 (25%) | n.v.t. (%) | 6 (20%) | 5 (18.5%) | 18 (20.2%) |
|  | 8 | 5 (25%) | 3 (25%) | n.v.t. (%) | 10 (33.3%) | 4 (14.8%) | 22 (24.7%) |
|  | 9 | 3 (15%) | 3 (25%) | n.v.t. (%) | 7 (23.3%) | 8 (29.6%) | 21 (23.6%) |
|  | 10 | 0 (0%) | 2 (16.6%) | n.v.t. (%) | 4 (13.3%) | 6 (22.2%) | 12 (13.5%) |
| In your opinion, how high is the trust between specialisms to talk openly about aggregated outcomes? (On a scale of 0-10) | 0 | 1 (5%) | 0 (0%) | 0 (0%) | 0 (0%) | 0 (0%) | 1 (0.8%) |
|  | 1 | 0 (0%) | 0 (0%) | 0 (0%) | 0 (0%) | 2 (7.4%) | 2 (1.6%) |
|  | 2 | 0 (0%) | 0 (0%) | 1 (2.9%) | 0 (0%) | 0 (0%) | 1 (0.8%) |
|  | 3 | 1 (5%) | 0 (0%) | 0 (0%) | 0 (0%) | 0 (0%) | 1 (0.8%) |
|  | 4 | 1 (5%) | 0 (0%) | 2 (5.7%) | 0 (0%) | 2 (7.4%) | 5 (4.0%) |
|  | 5 | 5 (25%) | 1 (8.3%) | 2 (5.7%) | 0 (0%) | 1 (3.7%) | 9 (7.3%) |
|  | 6 | 2 (10%) | 1 (8.3%) | 2 (5.7%) | 4 (13.3%) | 4 (14.8%) | 13 (10.5%) |
|  | 7 | 3 (15%) | 3 (25%) | 8 (22.9%) | 7 (23.3%) | 6 (22.2%) | 27 (21.8%) |
|  | 8 | 6 (30%) | 3 (25%) | 11 (31.4%) | 10 (33.3%) | 7 (25.9%) | 37 (29.8%) |
|  | 9 | 0 (0%) | 2 (16.6%) | 5 (14.3%) | 9 (30%) | 2 (7.4%) | 18 (14.5%) |
|  | 10 | 1 (5%) | 2 (16.6%) | 4 (11.4%) | 0 (0%) | 3 (11.1%) | 10 (8.1%) |
| **4. Leadership** |  |  |  |  |  |  |  |
| Different levels within an organisation are shown below. Please rank the levels in order of the extent to which a leadership role is taken to achieve an improvement cycle based on outcome indicators? (Highest priority) | Physicians | 16 (80%) | 12 (100%) | 17 (48.6%) | 16 (53.3%) | 20 (74.1%) | 81 (65.3%) |
|  | Medical society management | 0 (0%) | 0 (0%) | 15 (42.9%) | 8 (26.6%) | 2 (7.4%) | 25 (20.2%) |
|  | Hospital management | 0 (0%) | 0 (0%) | 0 (0%) | 0 (0%) | 1 (3.7%) | 1 (0.8%) |
|  | Nurses | 0 (0%) | 0 (0%) | 0 (0%) | 2 (6.6%) | 1 (3.7%) | 3 (2.4%) |
|  | Quality department | 2 (10%) | 0 (0%) | 3 (8.6%) | 1 (3.3%) | 2 (7.4%) | 8 (6.45%) |
|  | Board of directors | 1 (5%) | 0 (0%) | 0 (0%) | 3 (10%) | 1 (3.7%) | 5 (4.0%) |
|  | Staff board | 0 (0%) | 0 (0%) | 0 (0%) | 0 (0%) | 0 (0%) | 0 (0%) |
| Is there another level within the organisation which takes a leading role to realise an improvement cycle based on outcome indicators? | Yes | 4 (20%) | 4 (33.3%) | 3 (8.6%) | 4 (13.3%) | 8 (29.6%) | 23 (18.5%) |
|  | No | 16 (80%) | 8 (66.6%) | 32 (91.4%) | 26 (86.6%) | 19 (70.4%) | 101 (81.5%) |
| If yes, which level? | Quality department | 4 (100%) | 2 (50%) | 1 (33.3%) | 4 (100%) | 0 (0%) | 11 (8.9%) |
|  | Internal research committee | 0 (0%) | 0 (0%) | 1 (33.3%) | 0 (0%) | 0 (0%) | 1 (0.8%) |
|  | Research & Development | 0 (0%) | 1 (25%) | 0 (0%) | 0 (0%) | 2 (25%) | 3 (2.4%) |
|  | Infection prevention | 0 (0%) | 0 (0%) | 0 (0%) | 0 (0%) | 1 (12.5%) | 1 (0.8%) |
|  | Hospital hygiene department | 0 (0%) | 0 (0%) | 0 (0%) | 0 (0%) | 2 (25%) | 2 (1.6%) |
|  | Supporting services (such as pharmacy) | 0 (0%) | 0 (0%) | 1 (33.3%) | 0 (0%) | 0 (0%) | 1 (0.8%) |
|  | Other | 0 (0%) | 1 (25%) | 0 (0%) | 0 (0%) | 3 (37.5%) | 4 (3.2%) |
| Is there a explicit (non-)medical person with the responsibility for quality of care for patients? | No | 2 (10%) | 2 (16.6%) | 1 (2.9%) | 1 (3.3%) | 2 (7.4%) | 8 (6.45%) |
|  | Yes, the medical manager | 3 (15%) | 6 (50%) | 24 (68.6%) | 19 (63.3%) | 11 (40.7%) | 63 (50.8%) |
|  | Yes, but not the medical manager | 4 (20%) | 1 (8.3%) | 3 (8.6%) | 2 (6.6%) | 3 (11.1%) | 13 (10.5%) |
|  | Yes, not generic, but different specialists for different conditions | 7 (35%) | 1 (8.3%) | 2 (5.7%) | 2 (6.6%) | 8 (29.6%) | 20 (16.1%) |
|  | Other | 4 (20%) | 2 (16.6%) | 5 (14.3%) | 6 (20%) | 3 (11.1%) | 20 (16.1%) |
| **5. Infrastructure** |  |  |  |  |  |  |  |
| What percentage of data from the quality registration is recorded during regular care in the EHR? | 0 | 2 (10%) | 0 (0%) | 3 (8.6%) | 5 (16.6%) | 3 (11.1%) | 13 (10.5%) |
|  | 1 to 25 | 1 (5%) | 0 (0%) | 3 (8.6%) | 3 (10%) | 3 (11.1%) | 10 (8.1%) |
|  | 26 to 50 | 0 (0%) | 2 (16.6%) | 4 (11.4%) | 3 (10%) | 6 (22.2%) | 15 (12.1%) |
|  | 51 to 75 | 2 (10%) | 1 (8.3%) | 5 (14.3%) | 4 (13.3%) | 0 (0%) | 12 (9.7%) |
|  | 76 to 99 | 11 (55%) | 7 (58.3%) | 14 (40%) | 8 (26.6%) | 9 (33.3%) | 49 (39.5%) |
|  | 100 | 4 (20%) | 2 (16.6%) | 6 (17.1%) | 7 (23.3%) | 6 (22.2%) | 25 (20.2%) |
| How does the data get submitted to the quality registry? | Manual input into quality registration application (retyping) | 1 (5%) | 0 (0%) | 3 (8.6%) | 7 (23.3%) | 7 (25.9%) | 18 (14.5%) |
|  | Upload of template filled partly manually and partly by extraction from EHR | 10 (50%) | 8 (66.6%) | 11 (31.4%) | 9 (30%) | 9 (33.3%) | 47 (37.9%) |
|  | Upload of template filled by (almost) full extraction from EHR | 5 (25%) | 4 (33.3%) | 12 (34.3%) | 8 (26.6%) | 5 (18.5%) | 34 (27.4%) |
|  | Upload of template filled by (almost) full extraction from PDMS | 0 (0%) | 0 (0%) | 1 (2.9%) | 0 (0%) | 0 (0%) | 1 (0.8%) |
|  | Direct link between EHR and quality registration | 2 (10%) | 0 (0%) | 7 (20%) | 2 (6.6%) | 2 (7.4%) | 13 (10.5%) |
|  | Other | 2 (10%) | 0 (0%) | 1 (2.9%) | 4 (13.3%) | 4 (14.8%) | 11 (8.9%) |
| How is the data checked for quality before delivery? | Not | 0 (0%) | 0 (0%) | 2 (5.7%) | 1 (3.3%) | 1 (3.7%) | 4 (3.2%) |
|  | By a data manager | 16 (80%) | 9 (75%) | 15 (42.9%) | 9 (30%) | 11 (40.7%) | 60 (48.4%) |
|  | By a physician | 3 (15%) | 3 (25%) | 15 (42.9%) | 11 (36.6%) | 9 (33.3%) | 41 (33.1%) |
|  | Other | 0 (0%) | 0 (0%) | 3 (8.6%) | 9 (30%) | 6 (22.2%) | 18 (14.5%) |
| How often are data uploaded to the quality registry? | Not | 0 (0%) | 0 (0%) | 0 (0%) | 1 (3.3%) | 0 (0%) | 1 (0.8%) |
|  | Less than once a year | 0 (0%) | 0 (0%) | 0 (0%) | 0 (0%) | 0 (0%) | 0 (0%) |
|  | Once a year | 2 (10%) | 0 (0%) | 0 (0%) | 2 (6.6%) | 3 (11.1%) | 7 (5.6%) |
|  | Between once a year and quarterly | 8 (40%) | 2 (16.6%) | 1 (2.9%) | 4 (13.3%) | 2 (7.4%) | 17 (13.7%) |
|  | Quarterly | 6 (30%) | 8 (66.6%) | 8 (22.9%) | 15 (50%) | 6 (22.2%) | 43 (34.7%) |
|  | Between quarterly and monthly | 1 (5%) | 2 (16.6%) | 6 (17.1%) | 1 (3.3%) | 1 (3.7%) | 11 (8.9%) |
|  | Monthly | 1 (5%) | 0 (0%) | 17 (48.6%) | 2 (6.6%) | 7 (25.9%) | 27 (21.8%) |
|  | More often than monthly | 0 (0%) | 0 (0%) | 3 (8.6%) | 0 (0%) | 3 (11.1%) | 6 (4.8%) |
|  | Unknown | 2 (10%) | 0 (0%) | 0 (0%) | 5 (16.6%) | 5 (18.5%) | 12 (9.7%) |
| How high is your trust in the quality of data from your own hospital from the quality registration? (On a scale of 0-10) | 0 | 0 (0%) | 0 (0%) | 0 (0%) | 0 (0%) | 0 (0%) | 0 (0%) |
|  | 1 | 0 (0%) | 0 (0%) | 1 (2.9%) | 0 (0%) | 0 (0%) | 1 (0.8%) |
|  | 2 | 0 (0%) | 0 (0%) | 0 (0%) | 1 (3.3%) | 0 (0%) | 1 (0.8%) |
|  | 3 | 0 (0%) | 0 (0%) | 1 (2.9%) | 0 (0%) | 1 (3.7%) | 2 (1.6%) |
|  | 4 | 0 (0%) | 0 (0%) | 1 (2.9%) | 0 (0%) | 0 (0%) | 1 (0.8%) |
|  | 5 | 0 (0%) | 0 (0%) | 1 (2.9%) | 0 (0%) | 2 (7.4%) | 3 (2.4%) |
|  | 6 | 1 (5%) | 1 (8.3%) | 4 (11.4%) | 4 (13.3%) | 2 (7.4%) | 12 (9.7%) |
|  | 7 | 3 (15%) | 0 (0%) | 4 (11.4%) | 2 (6.6%) | 5 (18.5%) | 14 (11.3%) |
|  | 8 | 7 (35%) | 5 (41.6%) | 11 (31.4%) | 13 (43.3%) | 5 (18.5%) | 41 (33.1%) |
|  | 9 | 9 (45%) | 6 (50%) | 8 (22.9%) | 9 (30%) | 10 (37.0%) | 42 (33.9%) |
|  | 10 | 0 (0%) | 0 (0%) | 4 (11.4%) | 1 (3.3%) | 2 (7.4%) | 7 (5.6%) |
| How high is your trust in the quality of data from other hospitals from quality registration? (On a scale of 0-10) | 0 | 0 (0%) | 0 (0%) | 0 (0%) | 0 (0%) | 0 (0%) | 0 (0%) |
|  | 1 | 0 (0%) | 0 (0%) | 0 (0%) | 0 (0%) | 1 (3.7%) | 1 (0.8%) |
|  | 2 | 0 (0%) | 0 (0%) | 2 (5.7%) | 0 (0%) | 0 (0%) | 2 (1.6%) |
|  | 3 | 0 (0%) | 0 (0%) | 1 (2.9%) | 1 (3.3%) | 1 (3.7%) | 3 (2.4%) |
|  | 4 | 2 (10%) | 0 (0%) | 1 (2.9%) | 1 (3.3%) | 0 (0%) | 4 (3.2%) |
|  | 5 | 0 (0%) | 1 (8.3%) | 1 (2.9%) | 2 (6.6%) | 6 (22.2%) | 10 (8.1%) |
|  | 6 | 1 (5%) | 3 (25%) | 6 (17.1%) | 3 (10%) | 3 (11.1%) | 16 (12.9%) |
|  | 7 | 10 (50%) | 3 (25%) | 11 (31.4%) | 12 (40%) | 9 (33.3%) | 45 (36.3%) |
|  | 8 | 4 (20%) | 5 (41.6%) | 11 (31.4%) | 10 (33.3%) | 4 (14.8%) | 34 (27.4%) |
|  | 9 | 3 (15%) | 0 (0%) | 2 (5.7%) | 1 (3.3%) | 3 (11.1%) | 9 (7.3%) |
|  | 10 | 0 (0%) | 0 (0%) | 0 (0%) | 0 (0%) | 0 (0%) | 0 (0%) |
| **6. Staff** |  |  |  |  |  |  |  |
| Which of the following officer(s) are provided with time to implement an improvement cycle aimed at improving outcomes of care? | Medical specialists | 10 (43.47%) | 8 (57.1%) | 12 (30%) | 12 (27.9%) | 12 (37.5%) | 54 (35.5%) |
|  | Quality managers | 7 (30.4%) | 3 (21.4%) | 7 (17.5%) | 16 (37.2%) | 8 (25%) | 41 (27%) |
|  | Internal consultants | 2 (8.69%) | 0 (0%) | 1 (2.5%) | 1 (2.3%) | 2 (6.3%) | 6 (3.94%) |
|  | Medical managers | 2 (8.69%) | 0 (0%) | 4 (10%) | 3 (6.97%) | 4 (12.5%) | 13 (8.6%) |
|  | Specialism' managers | 0 (0%) | 0 (0%) | 2 (5%) | 0 (0%) | 1 (3.1%) | 3 (2.0%) |
|  | Statistician | 0 (0%) | 0 (0%) | 0 (0%) | 0 (0%) | 0 (0%) | 0 (0%) |
|  | Researcher | 0 (0%) | 0 (0%) | 1 (2.5%) | 0 (0%) | 1 (3.1%) | 2 (1.3%) |
|  | Departmental manager | 0 (0%) | 0 (0%) | 0 (0%) | 3 (6.97%) | 0 (0%) | 3 (2.0%) |
|  | Not applicable | 1 (4.34%) | 2 (14.3%) | 10 (25%) | 2 (4.7%) | 3 (9.4%) | 18 (11.8%) |
|  | Other | 1 (4.34%) | 1 (7.1%) | 3 (7.5%) | 6 (13.95%) | 1 (3.1%) | 12 (7.9%) |
| In total, how many hours per week do these individuals have available of this? | 0 | 0 (0%) | 2 (16.6%) | 6 (17.1%) | 1 (3.3%) | 1 (3.7%) | 10 (8.1%) |
|  | 1 to 8 | 17 (85%) | 8 (66.6%) | 21 (60%) | 20 (66.6%) | 18 (66.6%) | 84 (67.7%) |
|  | 8 to 16 | 3 (15%) | 1 (8.3%) | 4 (11.4%) | 4 (13.3%) | 4 (14.8%) | 16 (12.9%) |
|  | 16 to 24 | 0 (0%) | 1 (8.3%) | 0 (0%) | 3 (10%) | 3 (11.1%) | 7 (5.6%) |
|  | 24 to 32 | 0 (0%) | 0 (0%) | 0 (0%) | 1 (3.3%) | 0 (0%) | 1 (0.8%) |
|  | 36 to 40 | 0 (0%) | 0 (0%) | 2 (5.7%) | 1 (3.3%) | 2 (7.4%) | 5 (4.0%) |
|  | Not applicable | 0 (0%) | 0 (0%) | 2 (5.7%) | 0 (0%) | 0 (0%) | 2 (1.6%) |
| **7. Skills** |  |  |  |  |  |  |  |
| How many doctors are there in the hospital with clear expertise and affinity for data management and data analytics? | 0 | 1 (5%) | 0 (0%) | 4 (11.4%) | 3 (10%) | 3 (11.1%) | 11 (8.9%) |
|  | 1 | 3 (15%) | 2 (16.6%) | 12 (34.3%) | 11 (36.6%) | 5 (18.5%) | 33 (26.6%) |
|  | 2 to 5 | 16 (80%) | 9 (75%) | 19 (54.3%) | 15 (50%) | 18 (66.6%) | 77 (62.1%) |
|  | 6 to 20 | 1 (5%) | 1 (8.3%) | 0 (0%) | 1 (3.3%) | 1 (3.7%) | 4 (3.2%) |
|  | Not applicable | 0 (0%) | 0 (0%) | 0 (0%) | 0 (0%) | 0 (0%) | 0 (0%) |

*N/a = not applicable, CQR = clinical quality registration, HER = electronic health record, PDMS = Patient management system.*
